# A pancancer analysis of the oncogenic role of ZNRF2 in human tumours

**DOI:** 10.1111/jcmm.17900

**Published:** 2023-08-08

**Authors:** Fujie Shi, Yunfei Wu, Kai Wang, Jiafan Wang, Minghui Liu, Xinlei Sun

**Affiliations:** ^1^ State Key Laboratory of Natural Medicines, School of Life Science and Technology China Pharmaceutical University Nanjing China; ^2^ School of Life Sciences Nanjing University Nanjing China; ^3^ Division of Trauma and Surgical Intensive Care Unit Research Institute of General Surgery, Jinling Hospital, Medical School of Nanjing University Nanjing China

**Keywords:** cancer, hepatocellular carcinoma, immune infiltration, mutation, ZNRF2

## Abstract

Finding effective treatments for cancer requires a thorough understanding of how it develops and progresses. Recent research has revealed the crucial role that Zinc and ring finger 2 (ZNRF2) play in the progression of non‐small cell lung cancer (NSCLC) by controlling cell growth and death. However, a comprehensive analysis of ZNRF2's role in cancer as a whole has yet to be conducted. Our study sought to investigate the impact of ZNRF2 on diverse human tumours, as well as the molecular pathways involved, using databases such as TCGA (The Cancer Genome Atlas), GEO (Gene Expression Omnibus) and the Human Protein Atlas (HPA), as well as several bioinformatic tools. Our findings indicate that ZNRF2 is generally expressed at higher levels in tumours than in normal tissues, and in some cancers, its levels correlate positively with disease stage, potentially predicting a poor prognosis for patients. We also discovered genetic changes in ZNRF2 among cancer patients, as well as its relationship with cancer‐related fibroblasts, endothelial cells and immune cell infiltration. Additionally, we explored potential molecular mechanisms of ZNRF2 in tumours, finding that it increases in hepatocellular carcinoma (HCC) tissues and that inhibiting its expression through ZNRF2 siRNA can limit HepG2 cell proliferation. Overall, our study provides a comprehensive overview of ZNRF2's oncogenic roles across various cancers.

## 1 INTRODUCTION

Ubiquitin (Ub), discovered in 1975,^1^ is an evolutionarily conserved protein that post‐translationally marks proteins for degradation. Ubiquitination is the covalent attachment of polypeptide ubiquitin to lysine residues on target proteins. This process involves the addition of a small protein, ubiquitin or ubiquitin‐like proteins (UBLs), to target proteins for proteasome degradation or nondegradative signalling.^2^ Ubiquitination proceeds sequentially through a series of enzymatic reactions involving cooperation between activating (E1), binding (E2) and ligating (E3) enzymes.^3^ The cellular functions of ubiquitination span a broad spectrum, including proteolytic and nonproteolytic roles such as protein degradation,^4^, ^5^ DNA repair,^6^ the ER‐associated degradation (ERAD) pathway,^7^ receptor endocytosis,^8^ apoptosis,^9^ autophagy,^10^ as well as in cell and animal development.^11^ Otherwise, many proteins regulated by ubiquitylation control cellular processes relevant to tumorigeneses, such as cell cycle progression, apoptosis, receptor downregulation and gene transcription.^12^ E3 proteins recruit substrates to the ubiquitination machinery and play a crucial role in specifying for targeting proteins ubiquitination, so have substrate specificity.^13^ The E3 ligases are considered the most critical components of the ubiquitin conjugation machinery. Ubiquitin ligases fall into different classes based on their structural composition and mechanism of action, and they also characterize through the presence of either a HECT (homologous to E6AP C‐terminus), U‐box or RING (interesting new gene) domain.^12^ The activity of most E3s is specified by a RING domain, binding to an E2 ∼ ubiquitin thioester and activating discharge of its ubiquitin cargo.^14^

Zinc and ring finger 2 (ZNRF2) is a novel protein of the ZNRF proteins family containing a unique zinc finger‐RING finger motif in the C‐terminal, belonging to the E3 ligase.^13^ The membrane‐associated E3 ubiquitin ligase ZNRF2 activates and regulates the mTOR pathway.^13^ Hoxhaj et al.^15^ reported that ZNRF2 regulates the amino acid and the growth of cells. Zhang et al.^16^ detected significantly higher levels of ZNRF2 in NSCLC (non‐small cell lung cancer) tissues; also, high ZNRF2 levels were associated with poor prognosis in NSCLC patients. Overexpression of ZNRF2 increased NSCLC cell growth and inhibited apoptotic cell death. Hoxhaj et al.^17^ found that ZNRF2 is expressed highly in the entire CNS, has ubiquitin ligase activity, and may be crucial to establishing and maintaining neuronal transmission and plasticity. Gu et al.^18^ found that ZNRF2 plays a protective effect in CIRI (cerebral ischemia/reperfusion injury), and the underlying mechanism may be related to the inhibition of mTORC1‐mediated autophagy.^18^ However, previous studies about ZNRF2 are still limited to a few cancer types, and further exploration is needed to clarify its role in tumours.

To explore the function of ZNRF2 through various tumour types in a pancancer analysis, we take advantage of gene sequencing technology and bioinformatics, which provide new insights into tumour research.^19^ The projects, such as TCGA (The Cancer Genome Atlas) and ICGC (International Cancer Genome Consortium), can help people learn more about cancer.^20^ The TCGA is a public‐funded project that aims to catalogue and discover significant cancer‐causing genome alterations in large cohorts of over 30 human tumours through large‐scale genome sequencing and integrated multidimensional analyses.^19^ Tumour‐related functional genomics data sets can help to generate new cancer therapies, diagnostic methods and preventive strategies. In this study, we analysed the expression levels of ZNRF2 in various human tumours using various databases, such as TCGA, and we explored the ZNRF2 to survival status, genetic changes, immune infiltration and related molecular pathways in tumours. Furthermore, we used single‐cell sequencing data to analyse the expression and distribution of ZNRF2 in hepatocellular carcinoma (HCC) and further explore the function of ZNRF2 in the HepG2 cell line.

## 2 MATERIALS AND METHODS

### 2.1 Gene expression analysis

ZNRF2 expression data in cancer and adjacent normal tissues in TCGA database was downloaded from UCSC Xena (https://xenabrowser.net/).^21^ For some data without a comparison group, we used the GEPIA2^22^ (Gene expression profiling interactive analysis, version 2, http://gepia2.cancer‐pku.cn/#analysis) tool to obtain box plots of the GTEx database, under the settings of *p*‐value cutoff = 0.01, log_2_FC (fold change) cutoff =1, and ‘Match TCGA normal and GTEx data’. UALCAN^23^, ^24^ tool (http://ualcan.path.uab.edu/analysisprot.html) was used to analyse the cancer Omics data and the expression profiling of the ZNRF2 protein of the CPTAC (Clinical proteomic tumour analysis consortium) data set. GEPIA2 tool was used to analyse the ZNRF2 expression in different pathological stages of all TCGA cancers. The log2 [TPM (Transcripts per million) +1] transformed expression data were applied for the box or violin plots.^25^

### 2.2 Immunohistochemistry (IHC) staining

HPA^26^ (The Human Protein Atlas, https://www.proteinatlas.org) is an information database of protein expression patterns in normal human tissues, cells and cancers. In this study, IHC images of ZNRF2 protein expression in normal tissues and five tumour tissues, including BRCA (Breast invasive carcinoma), LUAD (Lung adenocarcinoma), STAD (Stomach adenocarcinoma), LIHC (Liver hepatocellular carcinoma) and UCEC (Uterine corpus endometrial carcinoma) were downloaded from the HPA.

### 2.3 Survival prognosis analysis

GEPIA2 provided customizable functions such as a tumour or normal tissue differential analysis, survival analysis, similar gene detection, correlation analysis and dimensionality reduction analysis. We used GEPIA2 to obtain the OS (Overall survival) and DFS (Disease‐free survival) significance map data and survival plots of ZNRF2 across all TCGA tumours. Cutoff‐high (50%) and cutoff‐low (50%) values were used as the expression thresholds for splitting the high‐expression and low‐expression cohorts.^25^

### 2.4 Genetic alteration analysis

The cBio Cancer Genomics Portal^27^, ^28^ (https://www.cbioportal.org) is an open access resource for interactive exploration of multidimensional cancer genomics data sets, providing access to more than 5000 tumour samples from 20 cancer studies. The cBioPortal tool was used to collect the data on alteration frequency, mutation type, mutated site information, CNA (Copy number alteration) and 3D (Three‐dimensional) protein structure across all TCGA tumours. Survival data, including OS and DFS, were compared for all the TCGA cancer types, with or without ZNRF2 genetic alteration. The Sangerbox tool^29^ was used to evaluate the relationship between ZNRF2 expression levels and TMB (tumour mutation load) or MSI (microsatellite instability).

### 2.5 Immune infiltration analysis

We used TIMER2^30^, ^31^, ^32^ (Tumour immune estimation resource, version 2, http://timer.cistrome.org/) and TISIDB^33^ (http://cis.hku.hk/TISIDB/) tools to analyse the relationship between ZNRF2 expression and tumour‐infiltrating lymphocytes (TILs), immunomodulators, chemokines in all TCGA tumours. Cancer‐associated fibroblast, neutrophil, endothelial cells and Tregs (Regulatory T cells) were selected for detailed analysis.

### 2.6 ZNRF2‐related gene enrichment analysis

The STRING^34^ website (https://string‐db.org/) was used to analyse the protein–protein interaction network. The main parameters were as follows: minimum required interaction score [‘Low confidence (0.150)’], meaning of network edges (‘evidence’), max number of interactors to show (‘no more than 50 interactors’ in 1st shell) and active interaction sources (‘experiments’).^35^ GEPIA2 was used to obtain the top 100 ZNRF2 positively associated genes based on the data sets of all TCGA tumours and normal tissues. The DAVID^36^, ^37^ (The Database for Annotation, Visualization and Integrated Discovery, https://david.ncifcrf.gov/summary.jsp) tool was used to GO and Kyoto Encyclopedia of Genes and Genomes (KEGG) analysis. The ggplot2 package was used for visualization.

### 2.7 HCC specimens

The use and handling of patient samples in this study were approved by the Medical Ethics Committee of the Jinling Hospital and Medical School of Nanjing University (Nanjing, China) (2019NZGKJ‐0137), and written informed consent was obtained from each participant. Four HCC patients who underwent primary surgical resection were enrolled in this study, and the paired HCC and adjacent noncancerous tissue (ANCT) were obtained from each patient. All the tissue sections were immediately frozen in liquid nitrogen at the time of surgery and stored at −80°C until analysis.

### 2.8 Single‐cell sequencing data

RNA single‐cell sequencing data of HCC patient was extracted from the GSE112271 in the GEO database, and included 21,143 cells from three tumoral regions in P13.^38^ The raw data was obtained, and processed by the Seurat (v 4.0.4)^39^ R package for downstream analysis. Low‐quality cells (minimum expression cells >3, gene numbers <200 and mitochondrial genes >10%) were filtered, and the rest of the cells were employed for bioinformatic analysis. After filtering, all data sets were combined through ‘merge’ function for further analysis, and the ‘ScaleData’ function was performed to ensure all genes was given equal weight. Clustering analysis was carried out with standard Seurat package procedures with a resolution at 0.5, and the identified clusters were visualized using t‐distributed Stochastic Neighbour Embedding (tSNE) of the principal components in Seurat. The clusters were annotated based on highly expressed genes, uniquely expressed genes and reported canonical cellular markers.

### 2.9 Cells

The cell lines HepG2 cells were purchased from the Shanghai Institute of Cell Biology (Shanghai, China), and cultured in 1640 medium of 10% inactivated foetal bovine serum (FBS) under 37°C. The HepG2 Cells were seeded in six‐well plates, and then were transfected the following day (~60%–80% confluence) using Lipofectamine 3000 (Thermo, USA) according to the manufacturer's instructions. For the transfection, 50 pmol siRNA (HIPPOBIO) per 10^5^ cells was used, and the cells were harvested 48 h after transfection for Western blotting. The siRNA used for knockdown ZNRF2 were as follows: hZNRF2 si‐1 sense: GAGGAUGUACUGAGUAAAGAUTT; hZNRF2 si‐2 sense: CCGCACAUGUUUGGAGGAUUUTT.

### 2.10 Western blotting

Tissues and cultured cells were lysed in a RIPA buffer (Beyotime, P0013B) with proteinase inhibitor cocktail (Beyotime, ST506), sonicated and centrifuged at 12000 × *g* for 10 min at 4°C, removing the deposition, and the protein concentration was determined by a BCA assay (Thermo Fisher, XE343717). Then the Western blot analysis was performed according to a standard method as described previously.^40^ The primary antibodies used for Western blot analysis were as follows: ZNRF2 antibody (1:1000; Proteintech, 20200‐1‐AP) and GAPDH antibody (1:1000; ABclonal, A19056). All the uncropped data of Western blotting in Figure S8.

### 2.11 CCK8

Cell Counting Kit‐8 (CCK‐8) reagent was used to measure the number of cells in the cell proliferation assay. About 12 h after the transfection of siRNA, 2 × 10^4^ cells were seeded in each well of 96‐well plates; 10 μL CCK‐8 reagent (CK04‐500, Dojindo, Japan) was added to each well to measure the cell proliferation index at a serial of specific time (0, 24, 48 and 72 h) according to the manufacturer's protocol. The absorbance value was measured by SpectraMax (Molecular Devices, USA) at 450 nm wavelength.

### 2.12 EdU

Cell proliferation was detected by Cell‐LightTM EdU Apollo 488 In Vitro Imaging Kit (RiboBio, C103103), according to the instructions. Briefly, cells were treated with 25 mM EdU for 2 h and then fixed by 4% paraformaldehyde. The cells were exposed to 1 × Apollo reaction mixture for 30 min. Finally, the cells were incubated with DAPI for 15 min and then imaged under fluorescence microscope (Nikon Ti2‐U). The signal of Edu + cells was analysed by image J software.

### 2.13 Statistical analysis

The R software (version 4.2.2) and Graphpad Prim software (version 9.4.0) were used to statistical analyses and visualization. Data are expressed as the means ± SEM. An unpaired *t* test and Wilcoxon rank‐sum test tested the significance of the difference between the two groups. The two‐way ANOVA analysis was used to examine the groups' statistical significance. One‐way ANOVA tested the significance of the difference between more than two groups. The significance of the correlation between the two groups was tested by Pearson and Spearman correlation analysis. The log‐rank test tested the significance of the prognostic values of the Kaplan–Meier curves. The Z‐values represent standard deviations from the median across samples for the given cancer type. A *p* value less than 0.05 was considered statistically significant. Significance was assumed for *p < 0.05, **p < 0.01 and ***p < 0.001.

## 3 RESULTS

### 3.1 Gene expression analysis data

In this study, we aimed to provide an analysis indicated a potential role of human ZNRF2 in tumours (NM_147128.4 for mRNA or NP_667339.1 for protein, Figure S1A). We found that the structure of ZNRF2 protein was highly conserved in different species (Figure S1B). The expression pattern of ZNRF2 in nontumour tissues and different cell lines was also analysed. Based on the HPA and GTEx data sets, although ZNRF2 was highly expressed in the testis, kidney and liver, it was comprehensively expressed in all tissues (normalized values were >1) with an overall low tissue specificity (Figure S2A). Based on the HPA data sets, we also found that plasmacytoid DC displayed the highest expression of ZNRF2, followed by T‐reg (Figure S2B).

**FIGURE 1:**
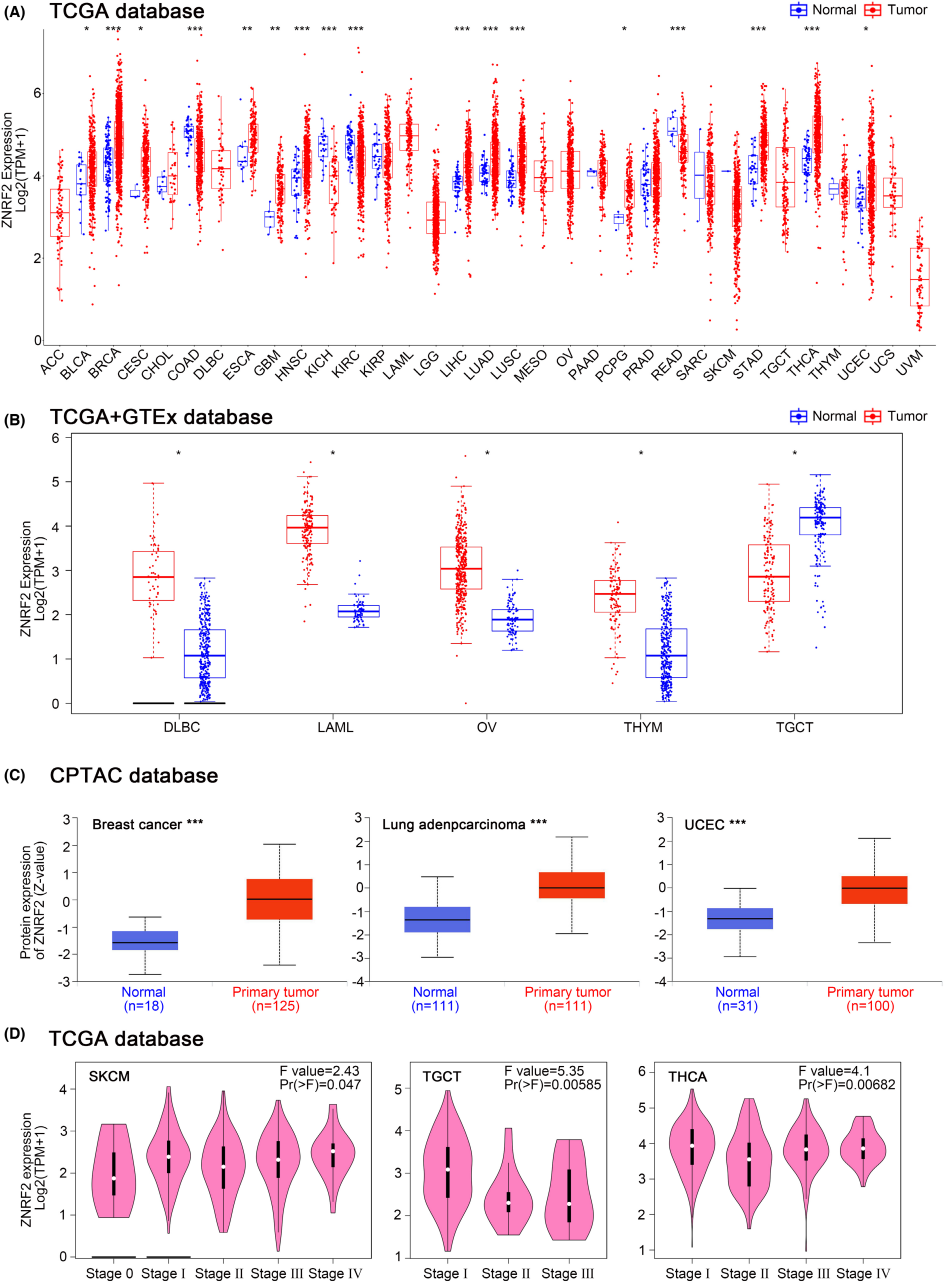
Expression and protein level of ZNRF2 in human tumours. (A) ZNRF2 expression in TCGA tumours and adjacent tissues. (B) ZNRF2 expression in DLBC, LAML, OV, THYM and TGCT compared with the corresponding normal tissues (GTEx database). (C) The total protein level of ZNRF2 in normal tissue and primary breast cancer, LUAD and UCEC. Protein data were extracted and analysed using CPTAC. (D) The expression of ZNRF2 in different clinical stages. Main pathological stages (stage I, stage II, stage III and stage IV) of SKCM, TGCT and THCA were assessed using TCGA data. The expression levels are shown as log_2_[TPM (Transcripts per million) + 1]. *p < 0.05. **p < 0.01. ***p < 0.001.

Next, we analysed the differential expression of ZNRF2 in tumours and adjacent normal tissues in the TCGA database. As shown in Figure 1A, the expression level of ZNRF2 in the tumour tissues of BRCA (Breast invasive carcinoma), HNSC (Head and neck squamous cell carcinoma), LIHC (Liver hepatocellular carcinoma), LUAD (Lung adenocarcinoma), LUSC (Lung squamous cell carcinoma), STAD (Stomach adenocarcinoma), THCA (Thyroid carcinoma) (*p* < 0.001), ESCA (Oesophageal carcinoma), GBM (Glioblastoma multiforme), (*p* < 0.01), BLCA (Bladder Urothelial Carcinoma), CESC (Cervical squamous cell carcinoma and endocervical adenocarcinoma), PCPG (Pheochromocytoma and Paraganglioma), UCEC (Uterine corpus endometrial carcinoma) (*p* < 0.05) was higher than corresponding control tissues. By contrast, ZNRF2 showed low expression in COAD (Colon adenocarcinoma), KICH (Kidney chromophobe), KIRC (Kidney renal clear cell carcinoma) and READ (Rectum adenocarcinoma) (*p* < 0.001). However, there was no difference in the expression of ZNRF2 in CHOL (Cholangiocarcinoma), KIRP (Kidney renal papillary cell carcinoma), PAAD (Pancreatic adenocarcinoma) and PRAD (Prostate adenocarcinoma) (*p* > 0.05) compared with corresponding control tissues.

**FIGURE 2:**
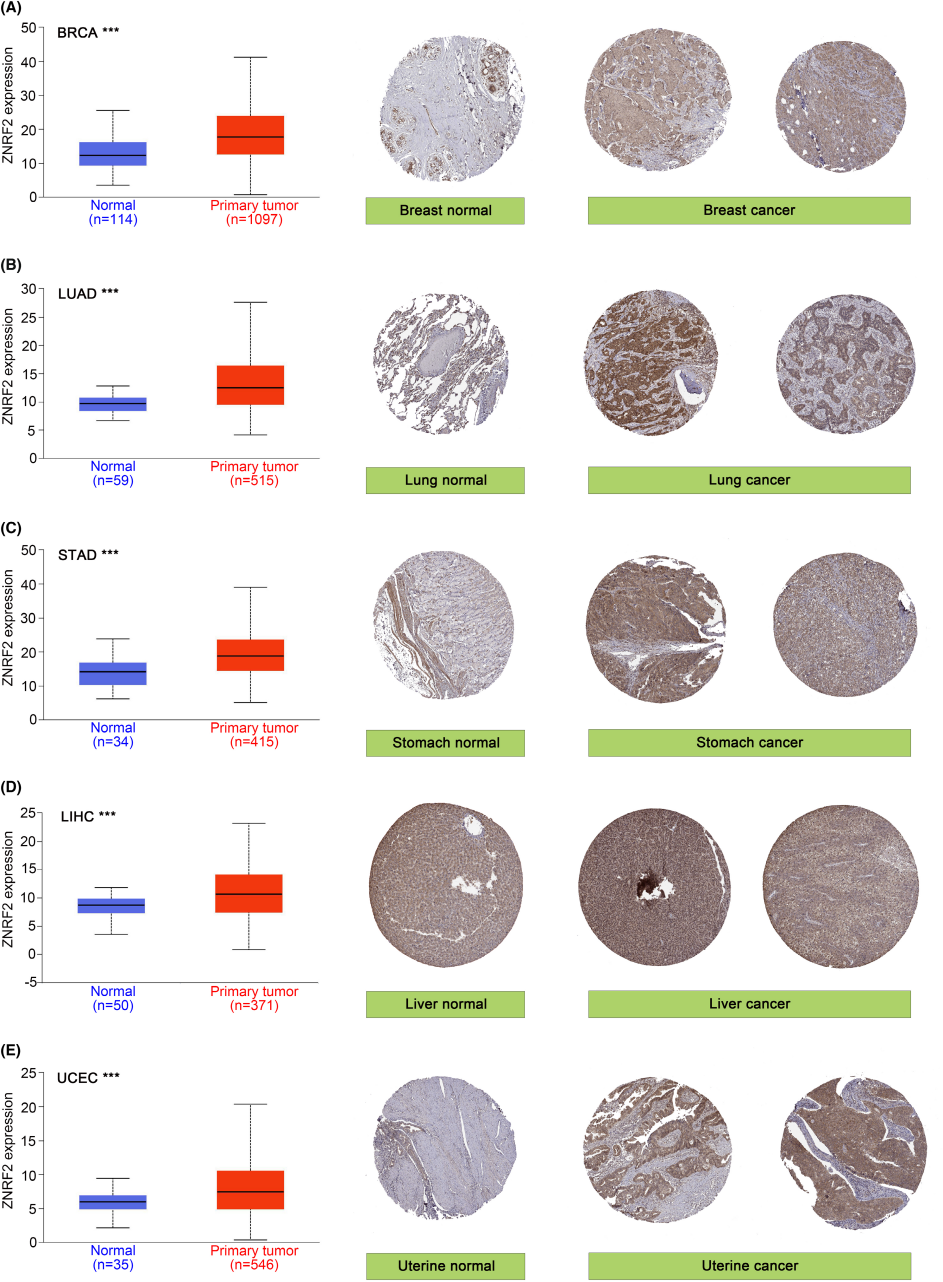
The differential expression of ZNRF2 in normal and tumour tissues (left) and immunohistochemistry images in normal (middle) and tumour (right) tissues (HPA). ZNRF2 was highly expressed in (A) BRCA, (B) LUAD, (C) STAD, (D) LIHC and (E) UCEC. *p < 0.05. **p < 0.01. ***p < 0.001.

For tumours without normal tissue data as control, we further used the GTEx data set to analyse the different expression level of ZNRF2 in tumours and normal tissues. As shown in Figure 1B, high expression of ZNRF2 was in DLBC (Lymphoid neoplasm diffuse large B‐cell lymphoma), LAML (Acute myeloid leukaemia), OV (Ovarian serous cystadenocarcinoma) and THYM (Thymoma) (*p* < 0.05), low expression of ZNRF2 was in TGCT (Testicular germ cell tumours) (*p* < 0.05). However, we did not get a significant difference for other tumours, such as ACC (Adrenocortical carcinoma), SARC (Sarcoma), SKCM (Skin cutaneous melanoma) and UCS (Uterine carcinosarcoma) (*p* > 0.05) (Figure S3). Overall, ZNRF2 was highly expressed in most tumour tissues. We further used the CPTAC database to assess differences in total ZNRF2 protein expression between tumour and normal tissues. We found that the expression levels of total ZNRF2 protein were higher in breast cancer, LUAD and UCEC tissues compared with normal tissues (Figure 1C). We also used the GEPIA2 tool to evaluate the correlation between ZNRF2 expression and the pathological stages of cancers. We observed significant differences in three tumours such as SKCM (*p* = 0.047), TGCT (*p* = 0.00585) and THCA (*p* = 0.00682) (Figure 1D). Nevertheless, in most tumours (*p* > 0.05), we did not find a significant difference (Figure S4). In addition, we analysed the results of IHC in the HPA database. As shown in Figure 2A–E, negative or moderate staining for ZNRF2 in normal tissues (breast, lung, stomach, liver and uterine) and moderate or vigorous positivity in the corresponding tumour tissues were in agreement with the results of the TCGA database. The expression of ZNRF2 was significantly higher in BRCA, LUAD, STAD, LIHC and UCEC (*p* < 0.001) than in the normal tissues that were used as controls.

### 3.2 Survival analysis data

**FIGURE 3:**
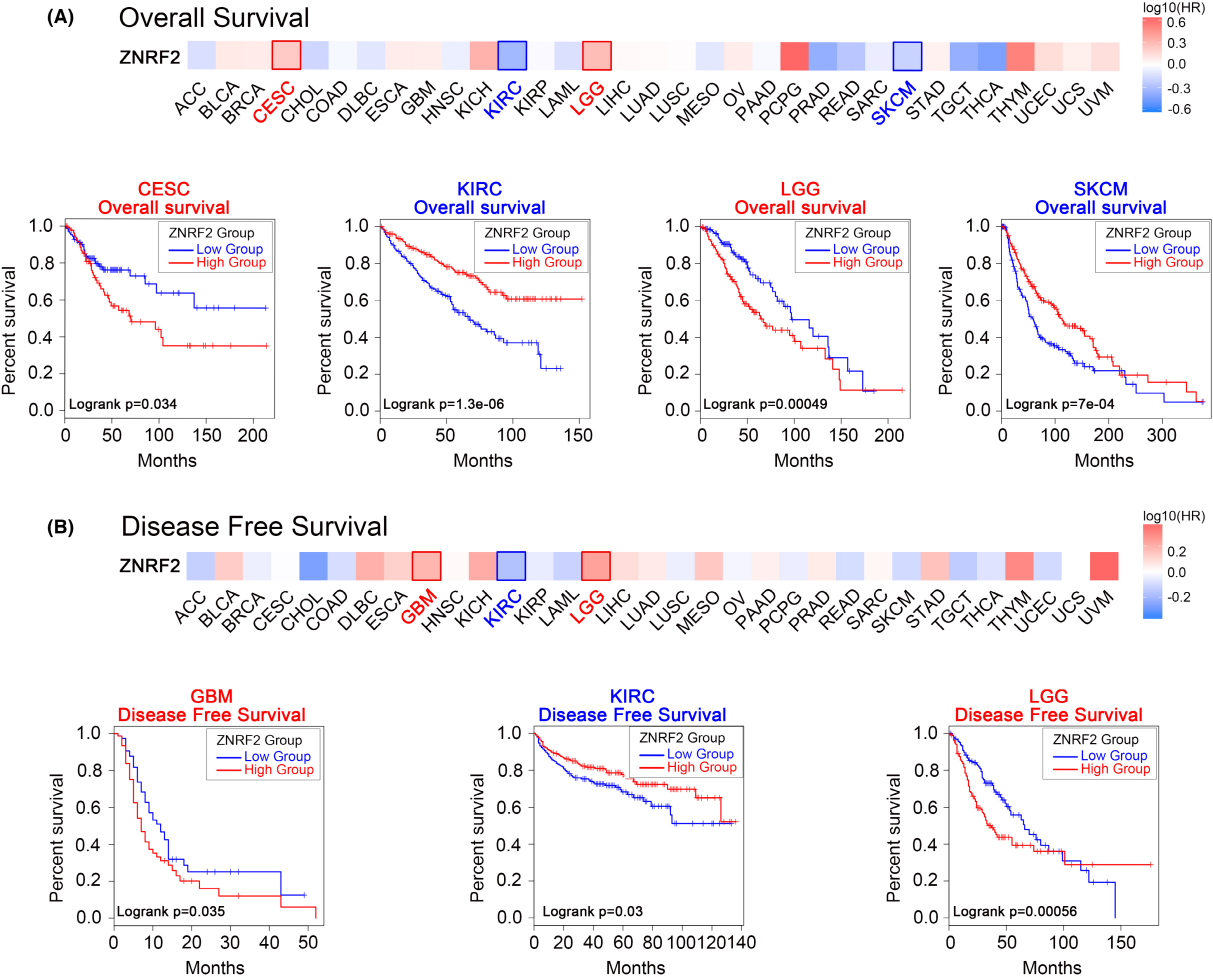
Relationship between ZNRF2 expression and survival curves (GEPIA2). Relationship between ZNRF2 expression levels and (A) Overall survival (OS), and (B) Disease‐free survival (DFS) curves in all TCGA tumours. The positive results of the survival map and Kaplan–Meier curves are listed.

Next, we used the TCGA and GEO data sets from the GEPIA2 tool to investigate the correlation between ZNRF2 expression and prognosis of different tumour patients. According to the expression levels of ZNRF2, the cancer cases were divided into high‐expression and low‐expression groups. As shown in Figure 3A, poor OS (overall survival) prognosis with high ZNRF2 expression in two tumour types: CESC (*p* = 0.034), LGG (*p* = 0.00049) and poor OS prognosis with low ZNRF2 expression in two tumour types: KIRC (*p* = 1.3e‐06), SKCM (*p* = 7e‐04). High expression of ZNRF2 was associated with poor prognosis of DFS (disease‐free survival) for cancers including GBM (*p* = 0.035) and LGG (*p* = 0.00056) (Figure 3B). In addition, in KIRC (*p* = 0.03), low expression of ZNRF2 was associated with poor prognosis of DFS.

Using the Kaplan–Meier mapping tool to analyse patient survival data, we found correlations between high levels of ZNRF2 and poor first progression (FP) (*p* = 0.0017) for lung cancer (Figure S5B). In addition, the correlation between low levels of ZNRF2 and poor OS (*p* = 0.00047), Postprogression survival (PPS) (*p* = 7.3e‐05) for lung cancer (Figure S5B), and poor OS (*p* = 7.2e‐08), FP (*p* = 1.5e‐06), PPS (*p* = 3.5e‐06) for gastric cancer (Figure S5C), and poor OS (*p* = 0.00014), DMFS (Distant metastasis‐free survival) (*p* = 0.011), PPS (*p* = 0.022), RFS (Relapse‐free survival) (*p* = 4.7e‐05) for breast cancer (Figure S5D) and poor OS (*p* = 0.005) for liver cancer (Figure S5E) prognosis were observed, respectively.

### 3.3 Genetic alteration analysis data

**FIGURE 4:**
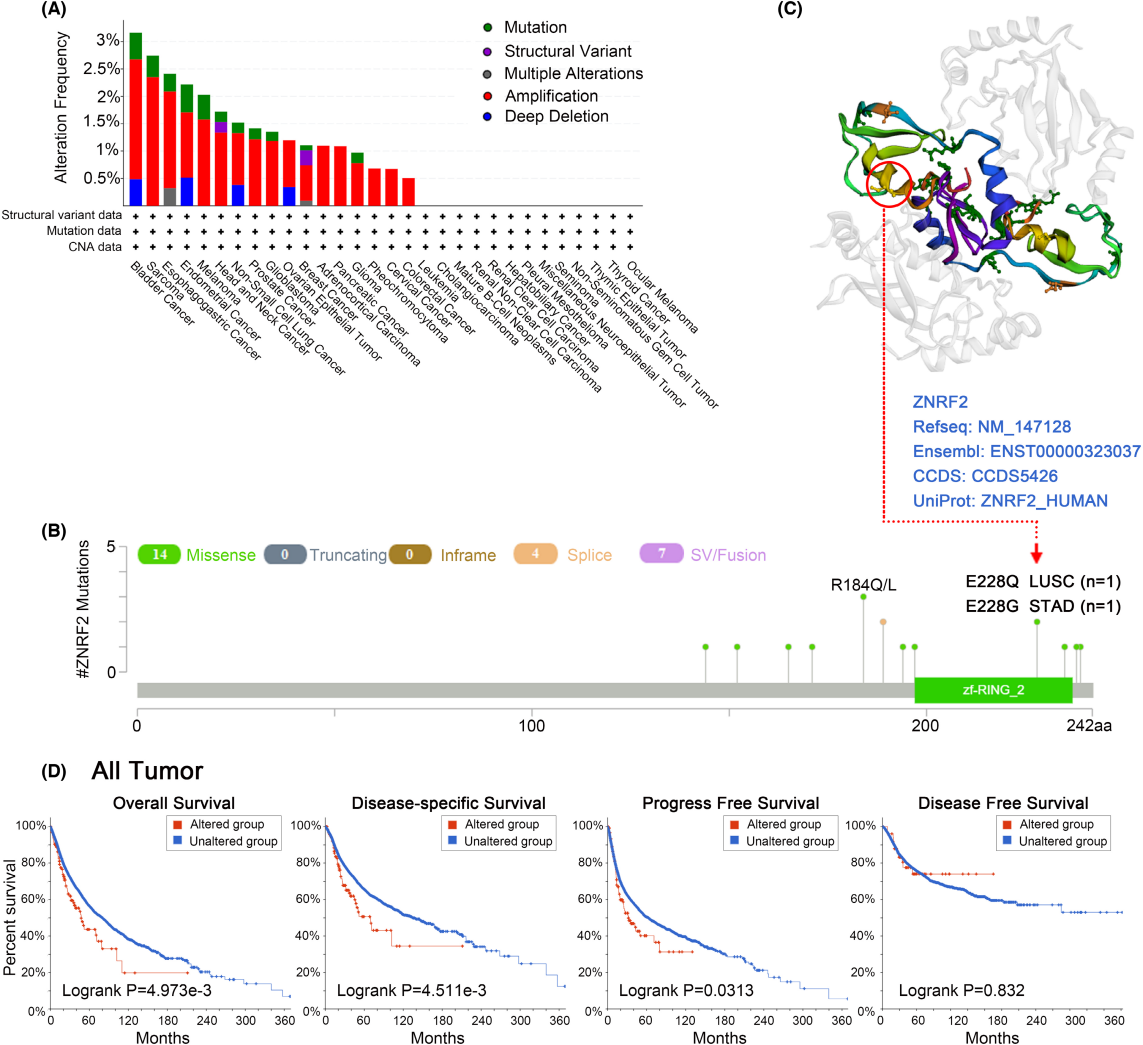
Mutation status of ZNRF2 in TCGA tumours (cBioPortal). (A) The alteration frequency with mutation type, (B) mutation site, (C) The 3D structure of ZNRF2 and mutation site, (D) the correlation between mutation status and OS (Overall survival), DSS (Disease‐specific survival), DFS (Disease‐free survival) and PFS (Progression‐free survival) in all TCGA tumours.

Due to mutations in genes are critical to the development of cancer, we further analysed the genetic alterations of ZNRF2 in tumour samples. As shown in Figure 4A, we found that the frequency (0.5%) of variants in ZNRF2 was highest in bladder cancer with ‘mutation’. Sarcoma had the highest incidence of ‘amplification’ type of CNA, with a frequency of ~2%. As shown in Figure 4B, we show other mutations and mutation sites of ZNRF2, and we obtained mutation sites visualized on the 3D structure of ZNRF2 (Figure 4C). We could observe a mutation at the E228 site in the 3D structure of the ZNRF2 protein (Figure 4C). Next, we investigated whether mutations in ZNRF2 affect the survival prognosis of cancer patients. As shown in Figure 4D, compared with patients without ZNRF2 mutation, the ZNRF2 mutation in patients had poor OS (*p* = 4.973e‐03), DSS (*p* = 4.511e‐03) and PFS (*p* = 0.0313) prognosis.

Next, we investigated the relationship between the expression of ZNRF2 and TMB (Tumour mutational burden) and MSI (Microsatellite instability). As shown in Figure S6A, we observed that ZNRF2 was significantly negatively correlated with TMB in two tumours: COAD (*p* < 0.001), COADREAD (Colon adenocarcinoma/Rectum adenocarcinoma oesophageal carcinoma) (*p* < 0.001). As shown in Figure S6B, we observed that ZNRF2 was significantly associated with MSI in 11 tumours. Among them, there were significant positive correlations in two types of tumours, such as STAD (*p* = 0.0075) and CHOL (*p* = 0.0039), and significant negative correlations in nine types of tumours, such as GBMLGG (Glioma) (*p* = 1.29e‐08), COAD (*p* = 1.45e‐11), COADREAD (*p* = 8.73e‐12), BRCA (*p* = 0.0047), PARD (*p* = 0.0006), HNSC (*p* = 8.3e‐06), TGCT (*p* = 0.012), UCS (*p* = 0.0002) and DLBC (*p* = 3.4e‐07). In conclusion, genetic changes in ZNRF2 are critical for developing the described tumours.

### 3.4 Protein phosphorylation analysis data

**FIGURE 5:**
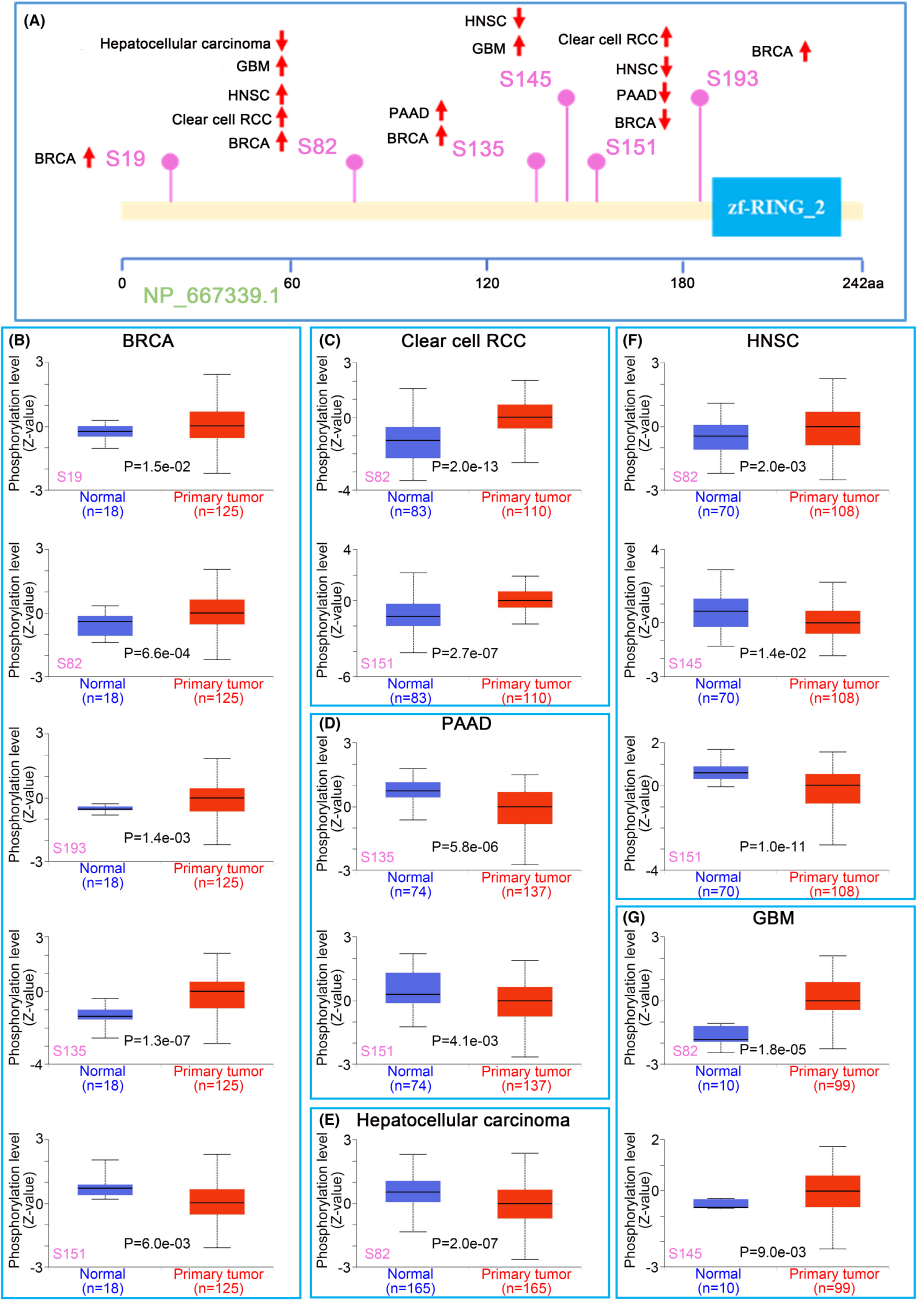
Phosphorylation analysis of ZNRF2 protein in different tumours. Based on the CPTAC data set, we analysed the expression level of ZNRF2 phosphoprotein (NP_667339.1, S19, S82, S135, S145, S151 and S193 sites) between normal tissue and primary tissue of selected tumours via the UALCAN. (A) The phosphoprotein sites with positive results are displayed in the schematic diagram of ZNRF2 protein. (B–G) The box plots for BRCA (B), clear cell RCC (C), PAAD (D), hepatocellular carcinoma (E), HNSC (F) and GBM (G).

We also compared the differences in ZNRF2 phosphorylation levels between normal and primary tumour tissues. The CPTAC data set analysed six types of tumours (BRCA, clear cell RCC, PAAD, hepatocellular carcinoma, HNSC and GBM) in Figure 5A, which showed that ZNRF2 had different phosphorylation sites. The S82 site of ZNRF2 exhibited higher phosphorylation levels in several primary tumour tissues, such as BRCA (*p* = 6.6e‐04), clear cell RCC (*p* = 2.0e‐13), HNSC (*p* = 2.0e‐03), GBM (*p* = 1.8e‐05) (Figure 5B,C,F,G), compared to normal tissues; however, the S151 site of ZNRF2 exhibited lower phosphorylation levels in several primary tumour tissues, such as BRCA (*p* = 6.0e‐03), PAAD (*p* = 4.1e‐03) and HNSC (*p* = 1.0e‐11) (Figure 5B,D,F). We also used the PhosphoNET database to analyse the phosphorylation of ZNRF2 identified by CPTAC and found that phosphorylation of the S82 site of ZNRF2 was associated with the mTOR signalling pathway^41^ (Table S1). This observation warrants further molecular testing to explore the potential role of ZNRF2 phosphorylation in tumorigenesis.

### 3.5 Immune infiltration analysis

**FIGURE 6:**
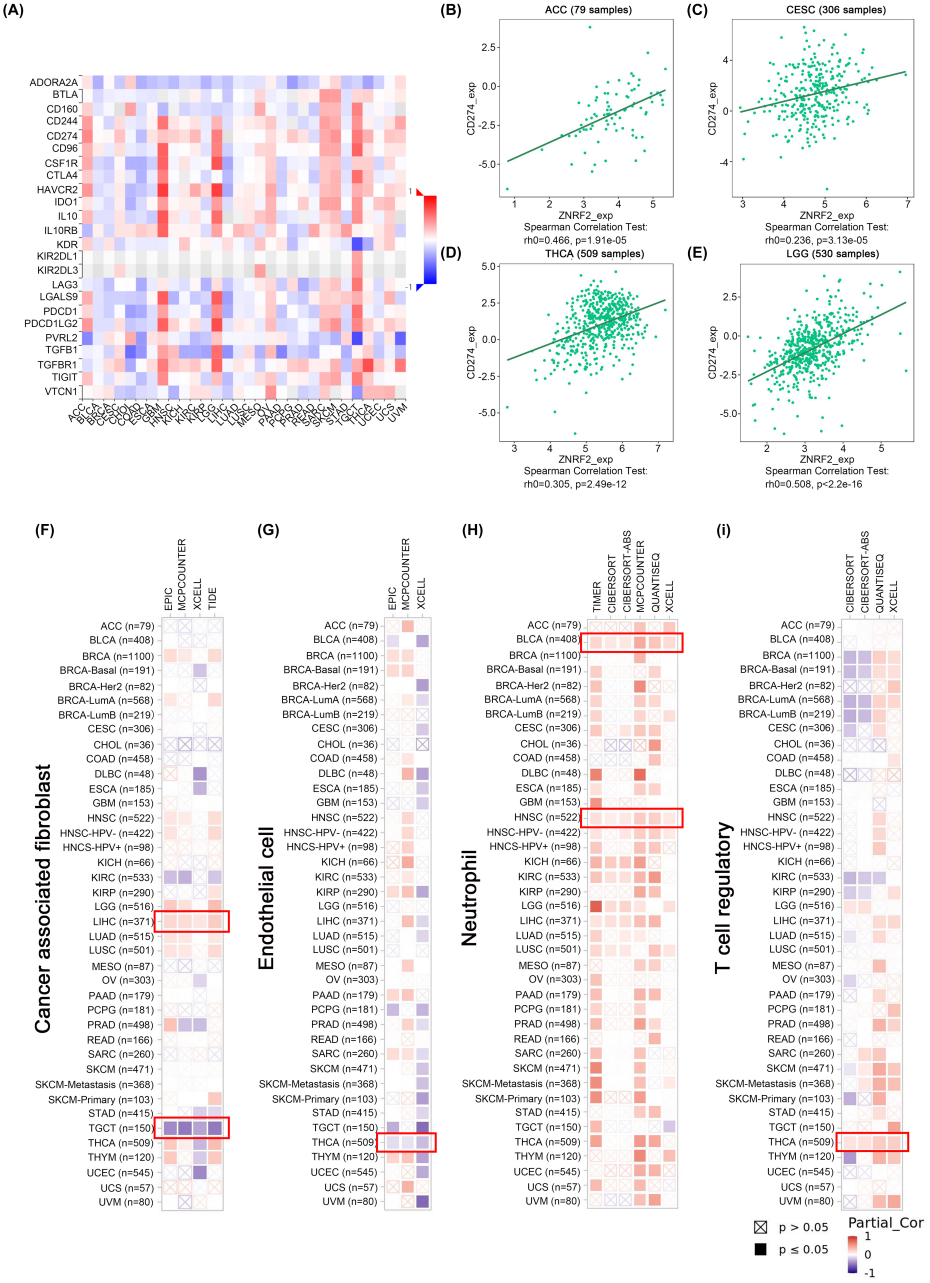
The correlation between ZNRF2 expression level and immune checkpoints, infiltration of cancer‐associated fibroblasts, endothelial cells, neutrophils and Tregs (TISIDB and TIMER2). (A) The correlation between ZNRF2 expression level and immune checkpoints, (B–E) The correlation between ZNRF2 expression level and CD274 in ACC, CESC, THCA and LGG. (F–I) The correlation between ZNRF2 expression level and infiltration of cancer‐associated fibroblasts (F), endothelial cells (G), neutrophils (H) and Tregs (I). The red colour indicates a positive correlation (0–1), and the blue colour represents a negative correlation (−1–0). The correlation with *p*‐value <0.05 is considered as statistically significant. Statistically nonsignificant correlation values are marked with a cross.

To investigate whether changes in the expression and genetic alterations of ZNRF2 affect the function of immune cells. We used the TISIDB tool to analyse the relationship between ZNRF2 expression and tumour‐infiltrating lymphocytes, immune‐stimulators, MHC molecules, chemokines and chemokine receptors (Figure S7). As shown in Figure 6A, we show the correlation of ZNRF2 expression levels with immune checkpoints. For example, the expression levels of ZNRF2 in ACC (Figure 6B) (*p* < 0.001), CESC (Figure 6C) (*p* < 0.001), THCA (Figure 6D) (*p* < 0.001) and LGG (Figure 6E) (*p* < 0.001) were significant positive correlation with CD274. Furthermore, we applied the TIMER, CiberSort, CiberSort‐ABS, TID, XCell, MCPCOUNTER, QUANTISEQ and EPIC algorithms to explore the correlation between the infiltration levels of different immune cells and endothelial cells in various tumour types and ZNRF2 expression. As shown in Figure 6F, the expression of ZNRF2 in LIHC (*p* < 0.05) was positively correlated with cancer‐associated fibroblasts, while the expression of ZNRF2 in TGCT (*p* < 0.05) was negatively correlated with cancer‐associated fibroblasts. However, the expression of ZNRF2 in THCA (*p* < 0.05) was negatively correlated with endothelial cell infiltration in Figure 5G and positively correlated with Treg infiltration (*p* < 0.05) (Figure 6I), moreover the expression of ZNRF2 in BLCA and HNSC (*p* < 0.05) was positively correlated with neutrophil infiltration (Figure 6H).

### 3.6 Enrichment analysis of ZNRF2‐related partners

**FIGURE 7:**
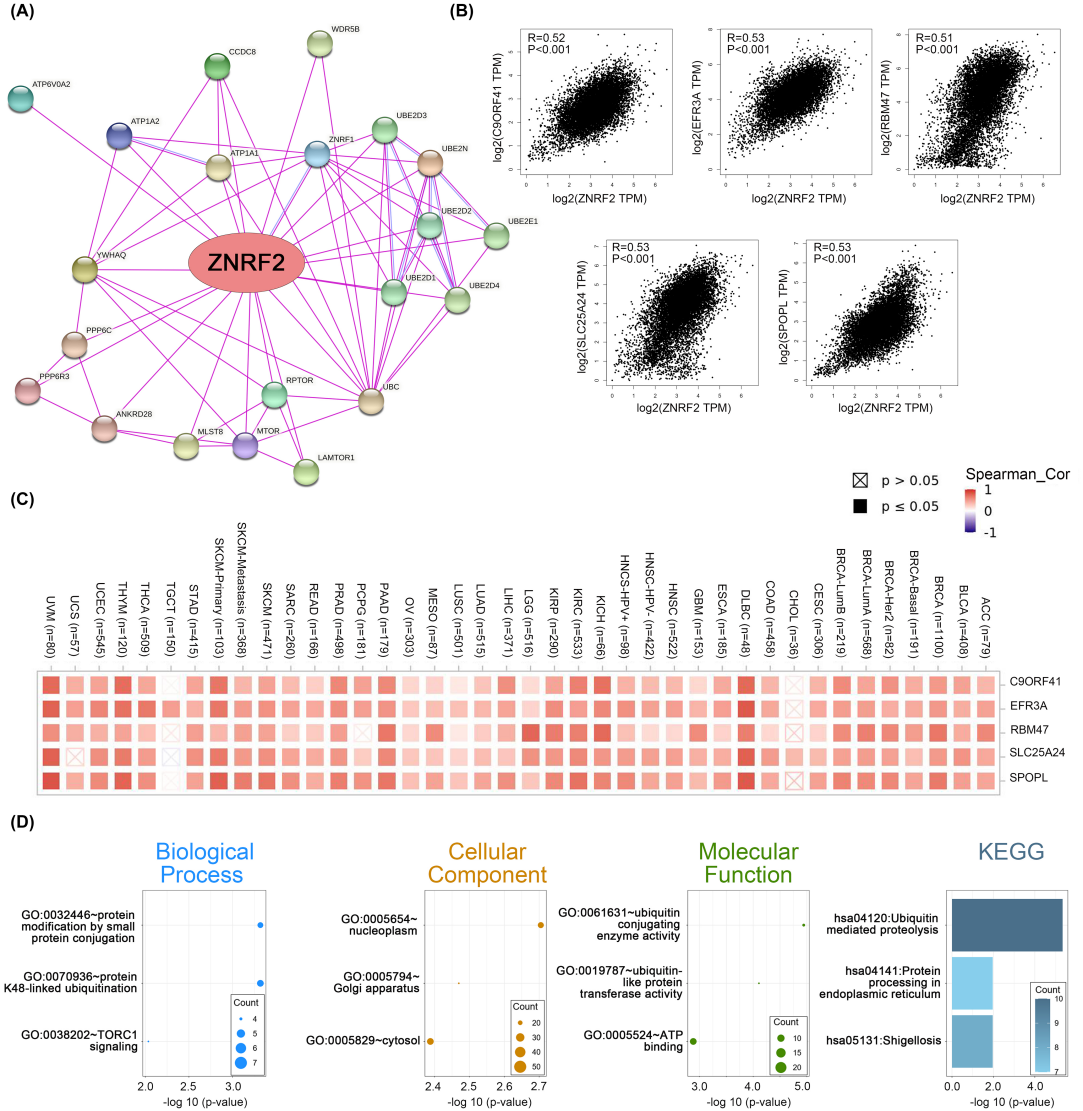
ZNRF2‐related gene enrichment and pathway analysis (STRING and GEPIA2). (A) STRING protein network map of experimentally determined ZNRF2‐interacting proteins. (B) Expression correlation between ZNRF2 and representative genes (SPOPL, SLC25A24, RBM47, EFR3A and C9ORF41) of the top ZNRF2‐correlated genes in TCGA database. (C) Heat map representation of the expression correlation data between ZNRF2 and SPOPL, SLC25A24, RBM47, EFR3A and C9ORF41 in the TCGA tumours. (D) GO biological process, GO cellular component, GO molecular function and Kyoto Encyclopedia of Genes and Genomes (KEGG).

To study the molecular mechanism of the ZNRF2 gene in tumorigenesis and development, we screened known ZNRF2‐interacting proteins using the STRING tool, which showed a network diagram of the action of ZNRF2 with interacting proteins (Figure 7A). Furthermore, we used the GEPIA2 tool to obtain the top 100 genes associated with ZNRF2 expression from the TCGA database. The expression of ZNRF2 has a strong positive correlation with SPOPL, SLC25A24, RBM47, EFR3A and C9ORF41 (Figure 7B) (*p* < 0.001). Similarly, heat map in most tumour types showed significant positive correlations of the above five genes with ZNRF2 (Figure 7C). Then, using the GEPIA2 to explore the biological function of ZNRF2, we evaluated the gene positively related to ZNRF2. We obtained 100 and 21 genes positively correlated with ZNRF2 in the TCGA and STRING databases, respectively, and then GO and KEGG analyzes were performed on the above gene lists using DAVID bioinformatics resources. As shown in Figure 7D, the most related biological processes with ZNRF2 included protein modification by small protein conjugation, protein K48‐linked ubiquitination and TORC1 signalling. The most relevant cell component of ZNRF2 was the nucleoplasm, and the following cell components were Golgi apparatus and cytosol. The molecular functions were ubiquitin conjugating enzyme activity, ubiquitin‐like protein transferase activity and ATP binding. The results of KEGG analysis were related to the ubiquitin‐mediated proteolysis, protein processing in endoplasmic reticulum and the Shigellosis.

### 3.7 Gene correlation analysis

**FIGURE 8:**
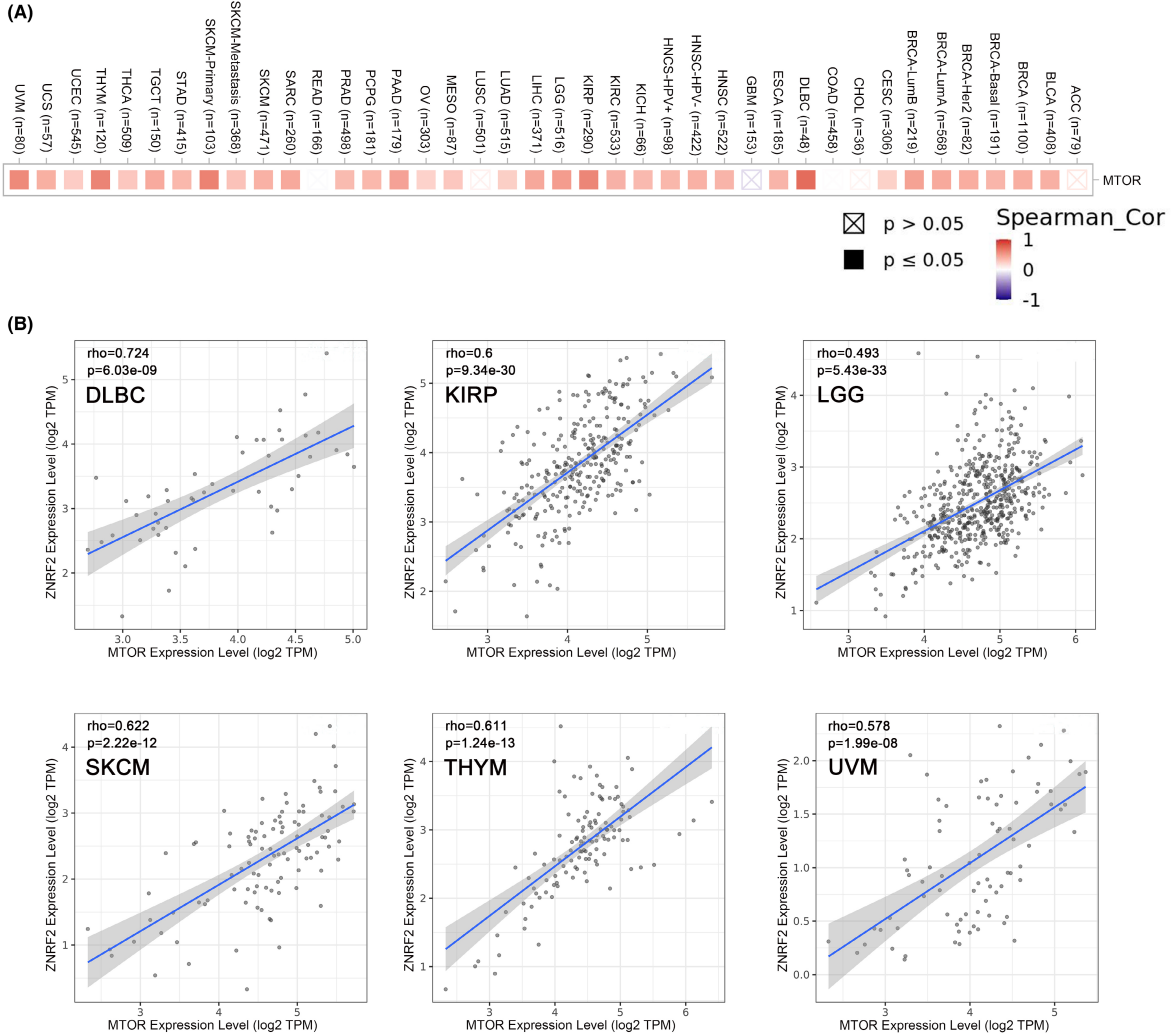
Correlation analysis of ZNRF2 expression levels with mTOR signalling pathway. (A) Heat map representation of the expression correlation data between ZNRF2 and MTOR in the TCGA tumours. (B) The correlation of ZNRF2 expression levels with mTOR in some tumours, such as DLBC, KIRP, LGG, SKCM, THYM and UVM.

Several studies have reported that ZNRF2 is associated with the rapamycin (mTOR) signalling pathway.^13^, ^15^, ^16^ The mTOR signalling pathway has a critical role in mammalian metabolism and physiology.^42^ The deregulated activity of mTOR is involved in many pathophysiological conditions, such as ageing, Alzheimer's disease, diabetes, obesity and cancer.^43^ Therefore, we hypothesized that ZNRF2 mediates the regulatory network of the mTOR signalling pathway and thus might influence the molecular mechanisms of tumour development. Next, we analysed the relationship between ZNRF2 and mTOR signalling pathway in TCGA tumours. As shown in Figure 8A, heat map in most tumour types showed significant positive correlations of the MTOR with ZNRF2. As shown in Figure 8B, we showed the correlation of ZNRF2 expression levels with MTOR in some tumours, such as DLBC, KIRP, LGG, SKCM, THYM and UVM (*p* < 0.001).

### 3.8 The expression of ZNRF2 was increased in HCC tissues and affected cell proliferation

**FIGURE 9:**
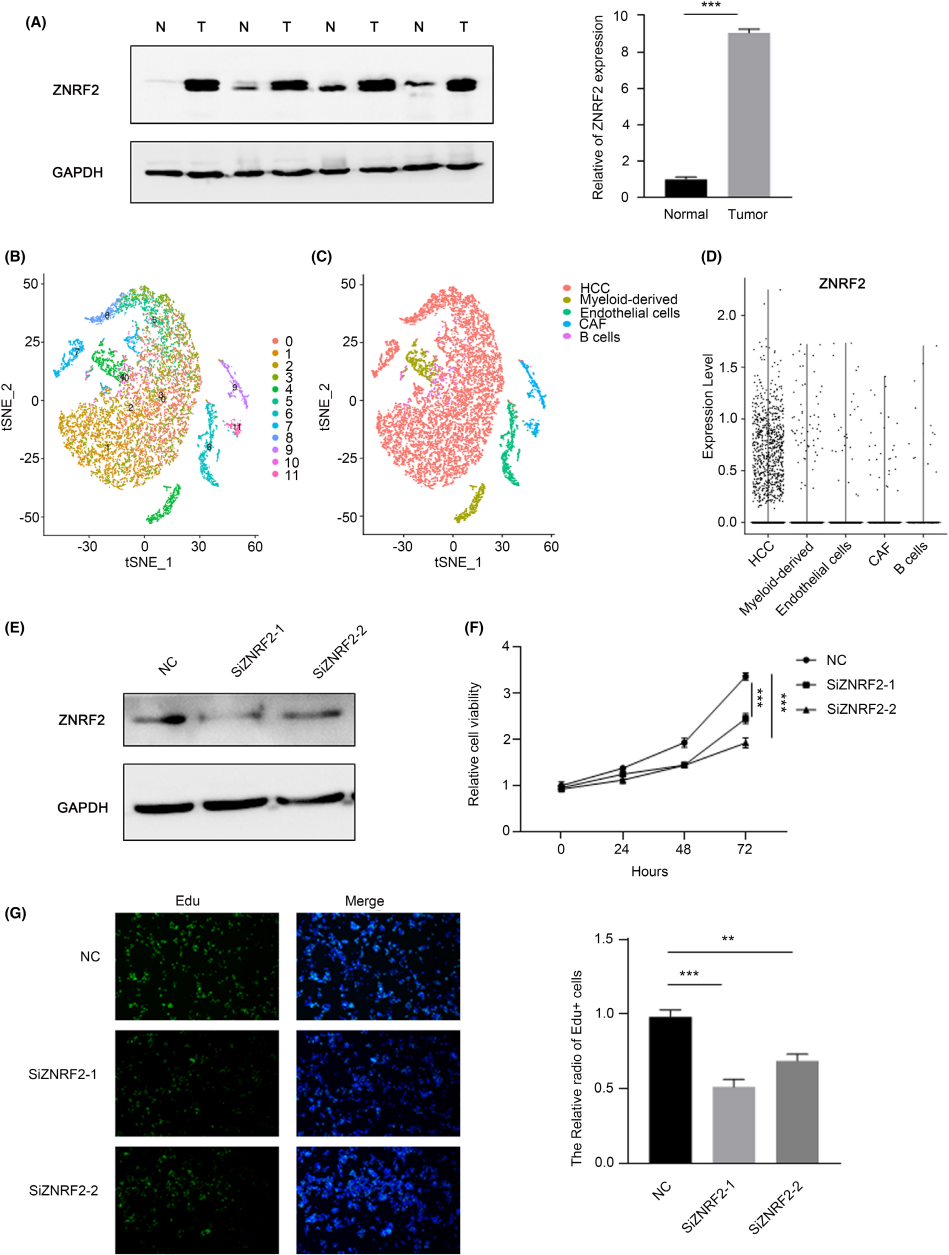
The expression of ZNRF2 was increased in HCC tissues and affected cell proliferation. (A) Western blotting of ZNRF2 in HCC tissues and ANCTs. N, normal tissues (ANCTs); T, tumour tissues (HCC tissues). (B) t‐SNE plots of single‐cell clusters. (C) t‐SNE plots of affiliation to cell lineage by gene expression. (D) The expression of ZNRF2 in different cell lineage. (E) ZNRF2 is knocked down by RNAi in HepG2 cells. (F) CCK8 assay of HepG2 cells treated with control, ZNRF2 siRNA‐1, −2. (G) EdU assay of HepG2 cells treated with control, ZNRF2 siRNA‐1, −2. **p < 0.01.***p < 0.001.

To summarize, our research reveals that ZNRF2 is significantly involved in the development of different types of tumours. In order to investigate how ZNRF2 affects the function of tumour cells, we conducted an examination of ZNRF2 expression patterns in four samples of hepatocellular carcinomas (HCC) tissues and four corresponding adjacent noncancerous tissues (ANCT) using Western blotting. The results showed that ZNRF2 was significantly upregulated in HCC tissues specimens compared with ANCT (Figure 9A). Thus, to explicitly examine which cell type mainly expressed ZNRF2 in HCC tissue, computing the t‐SNE plot and labelling cells based on the single‐cell data set GSE112271 that we obtained from the GEO database^38^ revealed an ecosystem of cells including hepatocytes (HCC) (ALB and FGG), cancer‐associated fibroblasts (CAF) (ACTA2), endothelial (KDR, VWF), myeloid‐derived (HLA‐DQB1, CD68) and sporadic B‐cells (IGJ, CD79A) (Figure 9B, C). We were further investigated the expression of ZNRF2 in the obtained cell population, as shown in Figure 9D, the ZNRF2 was dominant in HCC tumour cells instead of other type cells.

Previous studies have shown that the ZNRF2 affected the proliferation of NSCLC cells^16^ and osteosarcoma (OS),^44^ we examined that whether ZNRF2 played a same role in hepatocellular carcinoma. We knocked down ZNRF2 in HepG2 cells through the RNAi, and then performed the experimental verification using CCK8 and EdU. The results showed that ZNRF2 siRNA significantly inhibited the proliferation of HepG2 cells (Figure 9E, F, G).

## 4 DISCUSSION

Ub‐mediated cell signalling depends on degradative and nondegradative signals generated by various forms of Ub. The E3 ligase is essential for regulating ubiquitination network signalling. Dedicated E3 ligases tightly regulate the abundance of oncogenic proteins; by contrast, nondegradative Ub chains control the subcellular localization of signalling proteins and the assembly of signalling complexes, which, once deregulated, can lead to cancer.^45^ Zinc and ring finger 2 (ZNRF2) is a novel protein of the ZNRF proteins family containing a unique combination zinc finger‐RING finger motif in the C‐terminal, belonging to the E3 ligase.^13^

Previous studies have shown that the ZNRF2 involves a series of fundamental different physiological processes under multiple diseases, including cancer. The study has shown that ZNRF2 regulates the amino acid and the growth of cells.^15^ In non‐small cell lung cancer, ZNRF2 upregulates mTOR protein expression to promote cell proliferation and inhibit cell apoptosis.^16^ Li et al. suggested that hsa‐miR‐105 exerted tumour suppressor function by directly inhibiting the ZNRF2 signalling pathway.^46^ Liu et al.^47^ found that ZNRF2 can be a diagnostic molecular marker for cervical squamous cell carcinoma (CESC). In addition, Fang et al.^48^ indicate that lncRNA TTN‐AS1 worsens the course of tamoxifen‐resistant BC by regulating ZNRF2 via miR‐107 and activating the PI3K/AKT pathway.

Despite extensive research, the biological impact of ZNRF2 on normal and cancerous cells remains unclear. Our aim is to conduct a thorough analysis of ZNRF2 across different types of cancer. To accomplish this, we have utilized the TCGA, CPTAC and GEO databases to examine the gene expression and protein levels of ZNRF2 within tumours (Figures 1, 2). Additionally, we have investigated the genetic variations, immune response and molecular pathways associated with ZNRF2 in cancer.

The results of this study indicated that ZNRF2 is highly expressed in most tumours compared to the control group, which was further confirmed in hepatocellular carcinoma (Figure 9A–D). Previous studies have shown that overexpression of ZNRF2 promotes cancer cell growth and inhibits cancer cell apoptosis in NSCLC16^16^ and osteosarcoma^44^, which is consistent with the findings in liver cancer cells (HepG2) (Figure 9E–G). Additionally, it has been reported that ZNRF2 is associated with chemotherapy resistance in tumour cells^44^, ^46^ and our results suggest that ZNRF2 expression levels are correlated with the prognosis of patients in several tumours. Therefore, ZNRF2 may be a valuable diagnostic and therapeutic target for multiple tumours. Notably, in some tumour types such as KIRC, low expression of ZNRF2 was associated with a poor prognosis of disease‐free survival (*p* = 0.03) (Figure 3B). This finding may provide a novel clinical biomarker for predicting the disease‐free survival of KIRC patients and a potential therapeutic target.

Furthermore, our analysis revealed that the expression level of ZNRF2 is positively correlated with the immunosuppressive molecule CD274 in various tumours, and in THCA, it is positively correlated with Tregs (Figure 6). This suggested that targeting ZNRF2 may inhibit immunosuppressive cells in THCA, thereby reversing the immunosuppressive microenvironment and enhancing the ability of anticancer immune cells to kill tumour cells. Moreover, our study found that the phosphorylation level of ZNRF2 also exhibits abnormal changes in tumours, especially BRCA, KIRC, PAAD, LIHC, HNSC and GBM (Figure 5). Thus, further experimentation is necessary to investigate any potential correlation between alterations in ZNRF2 protein levels and phosphorylation levels and the immunosuppressive effects in the tumour microenvironment.

We have explored the potential molecular mechanisms that ZNRF2 may use to promote the development of tumours. According to some studies, ZNRF2 is linked to the mTOR signalling pathway.^15^, ^16^, ^18^ In our research, we discovered a positive correlation between the expression of ZNRF2 and mTOR in various types of tumours (as shown in Figures 7, 8). This indicated that the interaction between ZNRF2 and mTOR may be involved in tumour growth. Nonetheless, we still require further experiments to verify our results. In summary, our comprehensive analysis of ZNRF2 in pancancer cases reveals a high expression of ZNRF2 in many human tumours, along with a statistical correlation between ZNRF2 expression levels and patient survival, protein phosphorylation, immune cell infiltration, tumour mutational burden or microsatellite instability and mTOR signalling pathway. This contributes to our understanding of the critical role that ZNRF2 plays in tumour development.

## AUTHOR CONTRIBUTIONS

**Fujie Shi:** Conceptualization (supporting); data curation (equal); formal analysis (equal); methodology (lead); validation (lead); writing – original draft (equal). **Yunfei Wu:** Data curation (equal); formal analysis (equal); investigation (equal); software (equal). **Kai Wang:** Investigation (supporting); resources (equal); visualization (equal). **Jiafan Wang:** Data curation (supporting); formal analysis (supporting); investigation (supporting). **Minghui Liu:** Funding acquisition (equal); project administration (equal); supervision (equal); writing – original draft (supporting); writing – review and editing (supporting). **Xinlei Sun:** Conceptualization (lead); funding acquisition (lead); project administration (lead); supervision (equal); writing – original draft (equal); writing – review and editing (lead).

## FUNDING INFORMATION

This work was supported by grants from the National Natural Science Foundation of China (32000634), the Natural Science Foundation of the Jiangsu Province (BK20200572), the Postdoctoral Foundation of Jiangsu Province (2020Z263, 2020Z242).

## CONFLICT OF INTEREST STATEMENT

The authors declare no conflicts of interest.

## PATIENT CONSENT STATEMENT

The patients were informed and agreed to participate in this study.

## Supporting information


Appendix S1

## Data Availability

Publicly available data sets were analysed in this study. These data can be found here: https://xenabrowser.net/, https://www.ncbi.nlm.nih.gov/gds/, http://timer.cistrome.org/, https://www.proteinatlas.org/, http://gepia2.cancer‐pku.cn/, https://www.cbioportal.org/, http://cis.hku.hk/TISIDB/, https://string‐db.org/, https://github.com/sfj318/jcmm_code.git.
