# Supplementary material for: A pancancer analysis of the oncogenic role of ZNRF2 in human tumours
Source: J Cell Mol Med. 2023 Aug 8;27(21):3296–312. doi: 10.1111/jcmm.17900 (PMC10623518; doi:10.1111/jcmm.17900)
Supplement: Supplementary file 1 — Appendix S1 [file JCMM-27-3296-s001.docx]

**A Pan-Cancer Analysis of the Oncogenic Role of ZNRF2 in Human Tumors**

Fujie Shi^#1,2^, Yunfei Wu^#1^, Kai Wang^#3^, Jiafan Wang^1^, Minghui Liu^*1^, Xinlei Sun^*1^

1 State Key Laboratory of Natural Medicines, School of Life Science and Technology, China Pharmaceutical University, 639 Longmian Avenue, Nanjing, Jiangsu, 211198, China;

2 School of Life Sciences, Nanjing University, 163 Xianlin Avenue, Nanjing, Jiangsu, 210023, China;

3 Division of Trauma and Surgical Intensive Care Unit, Research Institute of General Surgery, Jinling Hospital, Medical School of Nanjing University, Nanjing, Jiangsu, 210002, China.

#These authors contributed equally to this work

∗Corresponding author

Minghui Liu, State Key Laboratory of Natural Medicines, School of Life Science and Technology, China Pharmaceutical University, Nanjing 211198, China.minghuiliu@cpu.edu.cn

Xinlei Sun, State Key Laboratory of Natural Medicines, School of Life Science and Technology, China Pharmaceutical University, Nanjing 211198, China. xsun@cpu.edu.cn

**Running title:** Role of ZNRF2 in Tumor

**Supplementary Figure 1**

**Supplementary Figure 2**

**Supplementary Figure 3**

**Supplementary Figure 4**

**Supplementary Figure 5**

**Supplementary Figure 6**

**Supplementary Figure 7**

**Supplementary Figure 8**

**Table S1.**


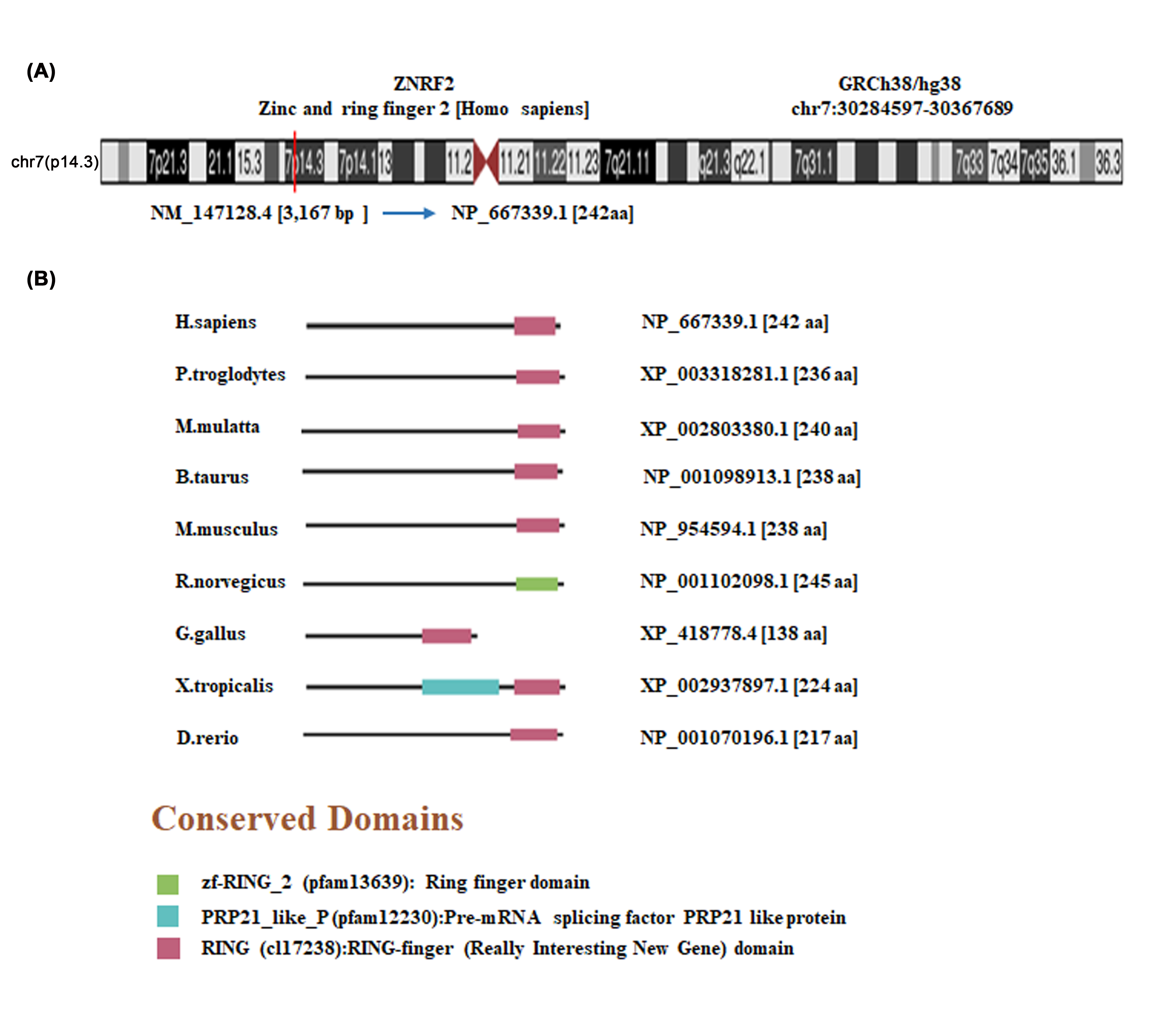


**Supplementary Figure 1 Structural characteristics of ZNRF2 in different species. (A)** Genomic location of human ZNRF2, **(B)** Conserved domains of ZNRF2 protein among different species.


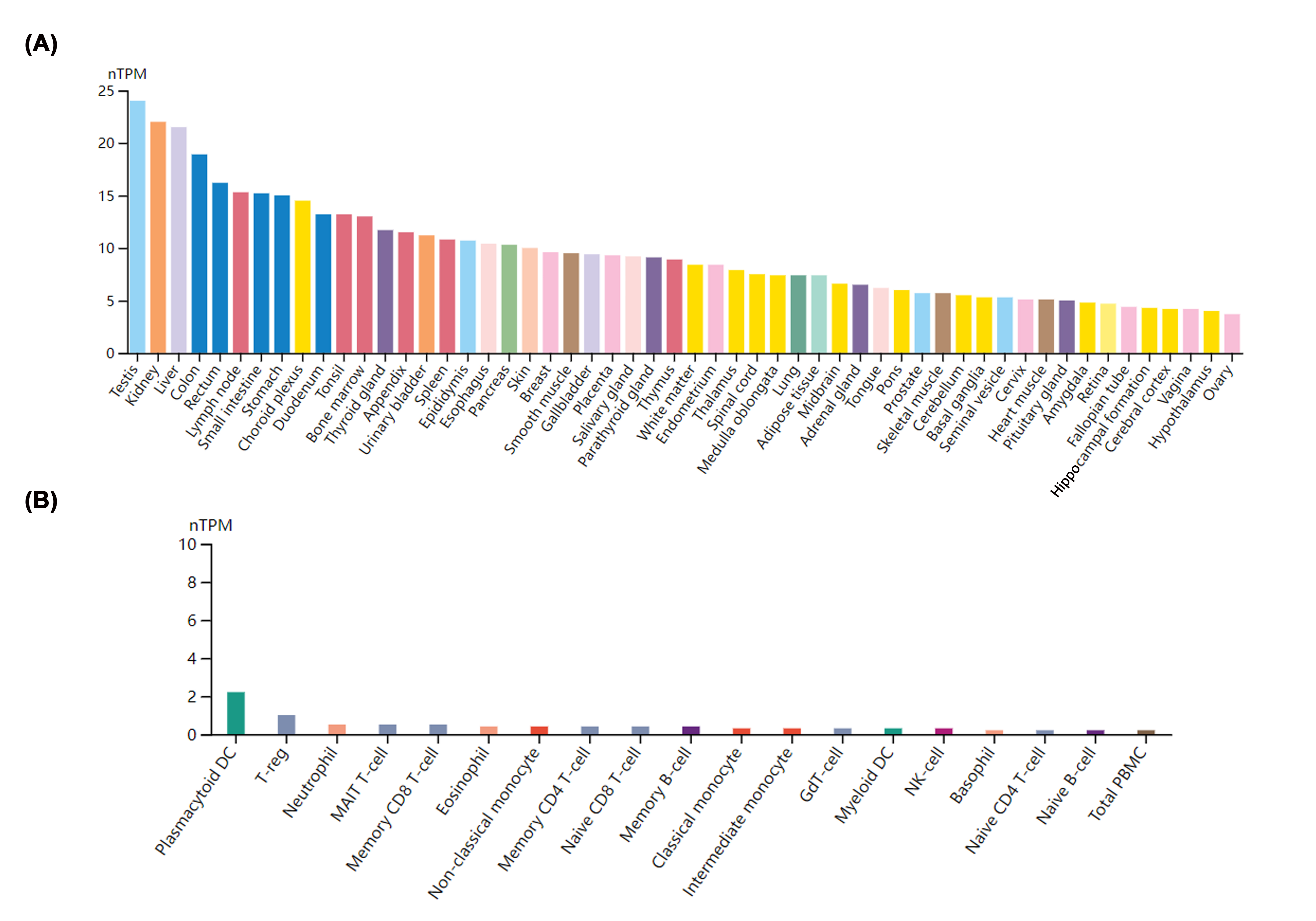


**Supplementary Figure 2 Expression level of ZNRF2 in different tissues and blood cells in the normal physiological state.** **(A)** the expression of the ZNRF2 gene in different tissues in the consensus dataset of HPA and GTEx dataset, **(B)** in different blood cells in the HPA dataset.


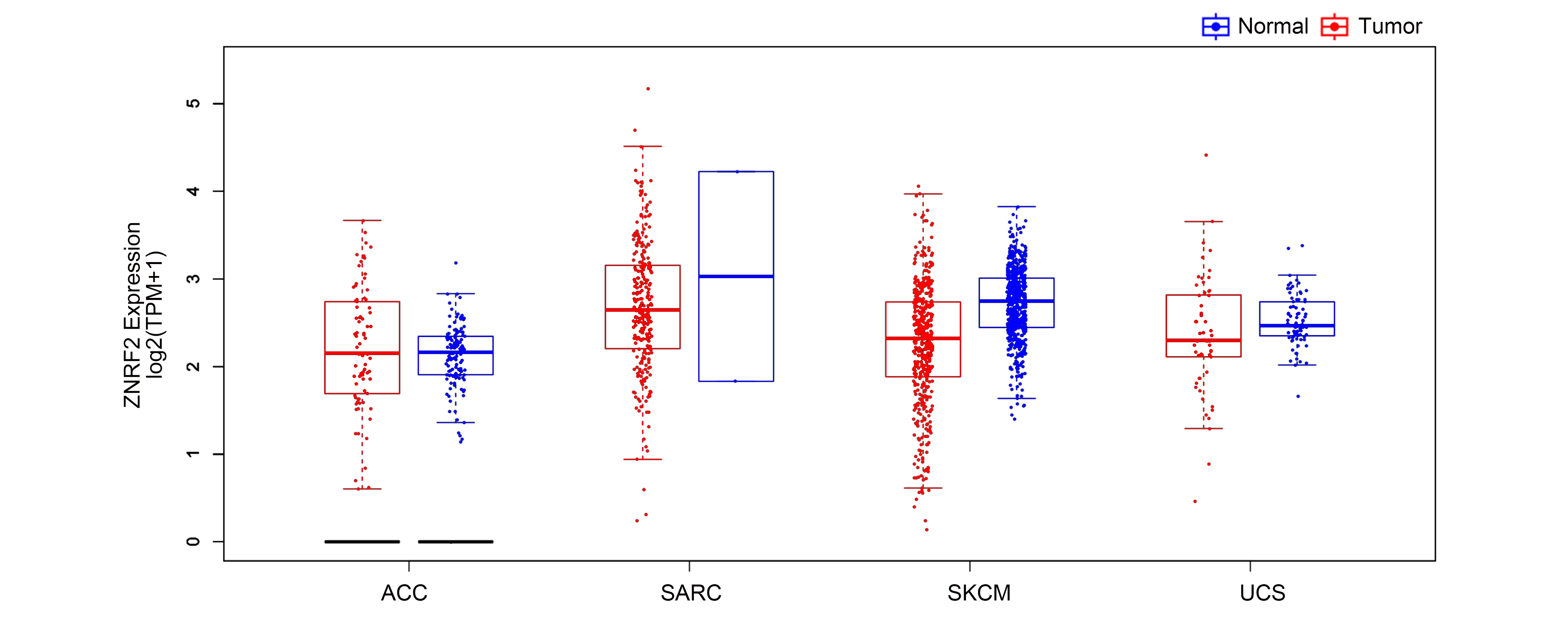


**Supplementary Figure 3 Expression level of the ZNRF2 gene in different tumors.** The expression statuses of the ZNRF2 gene in ACC, SARC, SKCM, and UCS in TCGA (P>0.05) project were compared with the corresponding normal tissues of the GTEx databases.


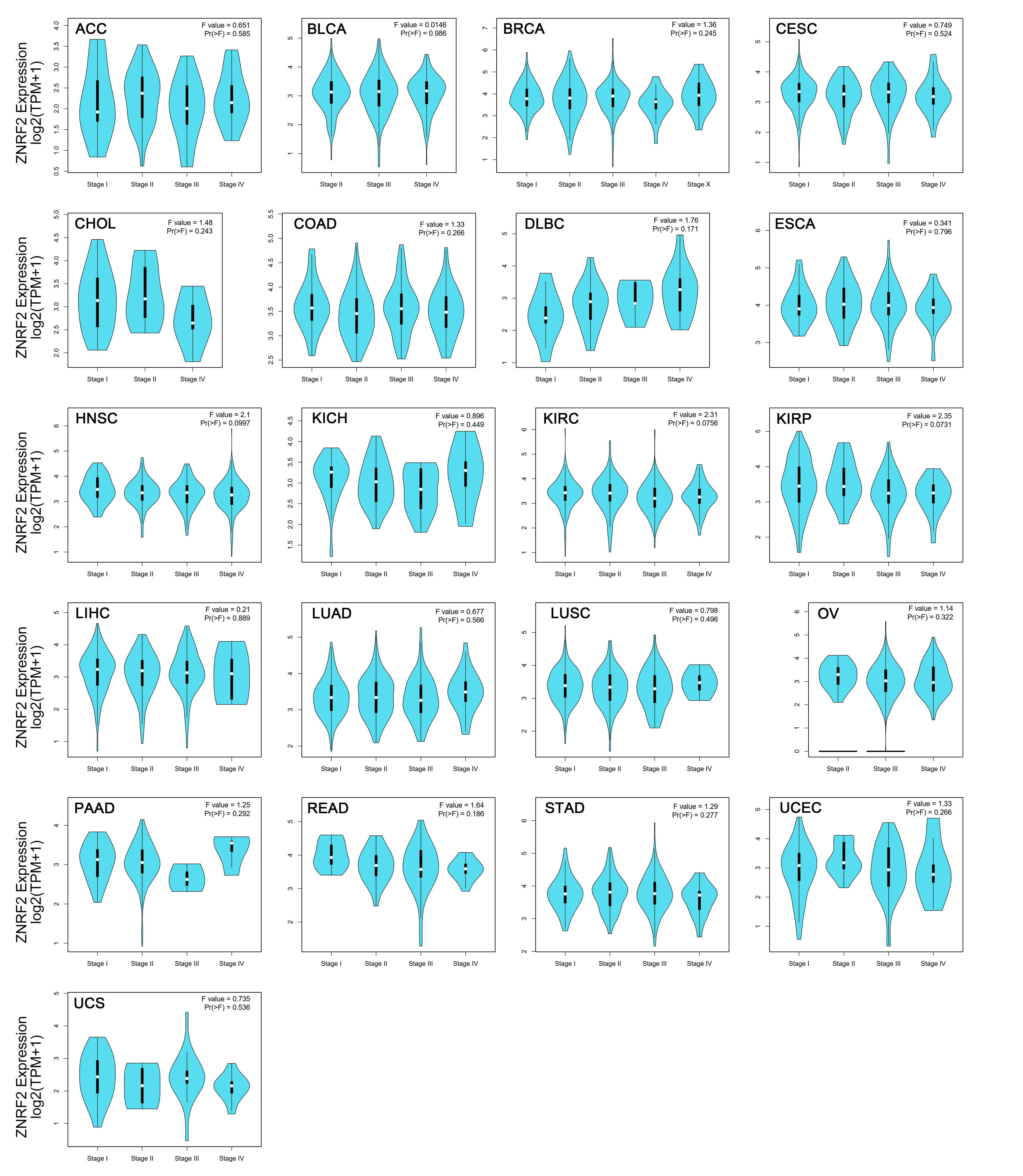


**Supplementary Figure 4 Expression level of the ZNRF2 gene in different TCGA tumors pathological stages.** Including ACC, BLCA, BRCA, CESC, CHOL, COAD, DLBC, ESCA, HNSC, KICH, KIRC, KIRP, LIHC, LUAD, LUSC, OV, PAAD, READ, STAD, UCEC, UCS (P>0.05).


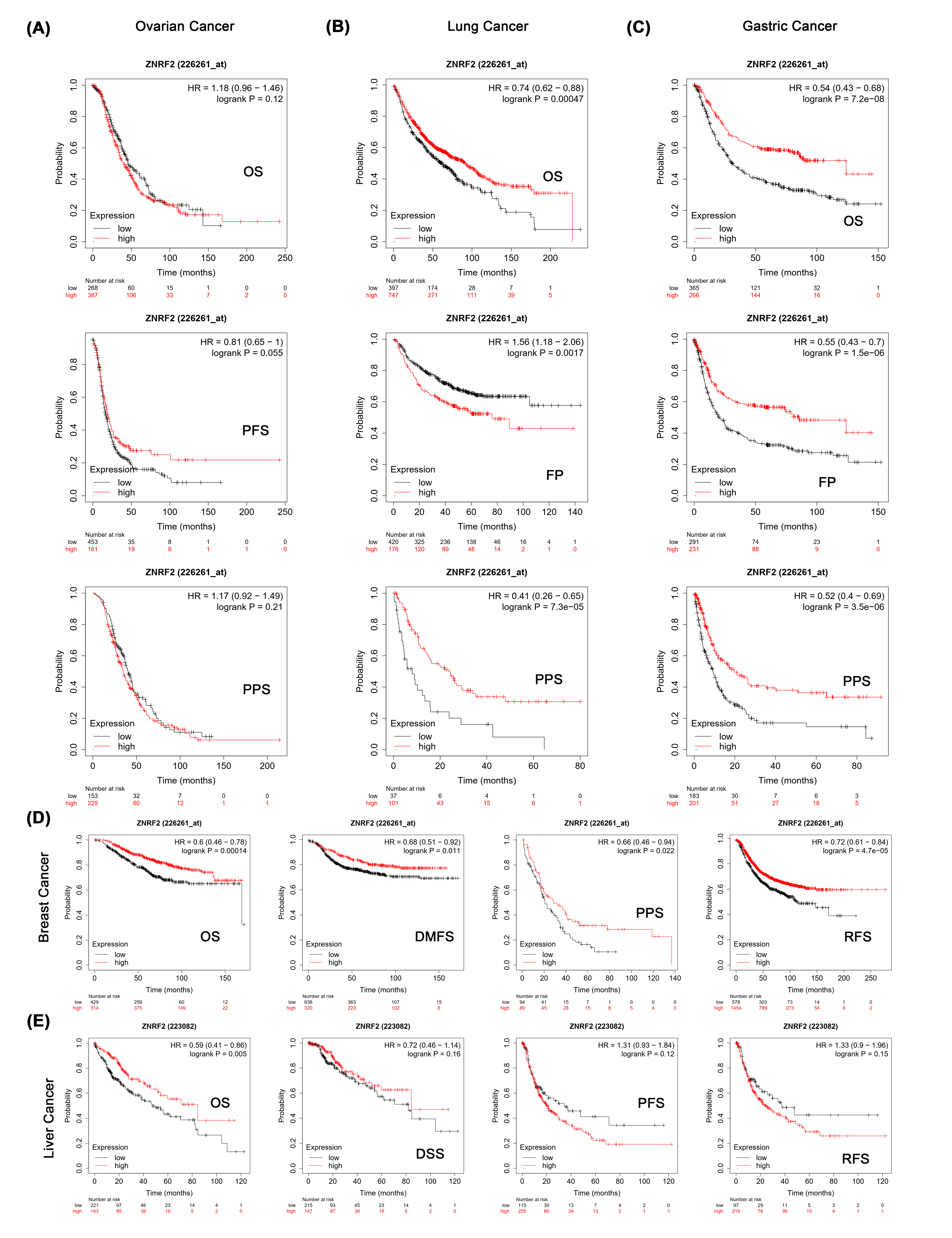


**Supplementary Figure 5 ZNRF2 expression level and tumor prognosis.** We analyzed the relationship between ZNRF2 expression levels and survival curves (OS, DMFS, RFS, PFS, PPS, FP, and DSS) using the Kaplan-Meier plotter. **(A)** Ovarian cancer, **(B)** Lung cancer, **(C)** Gastric cancer, **(D)** Breast cancer, **(E)** Liver cancer.


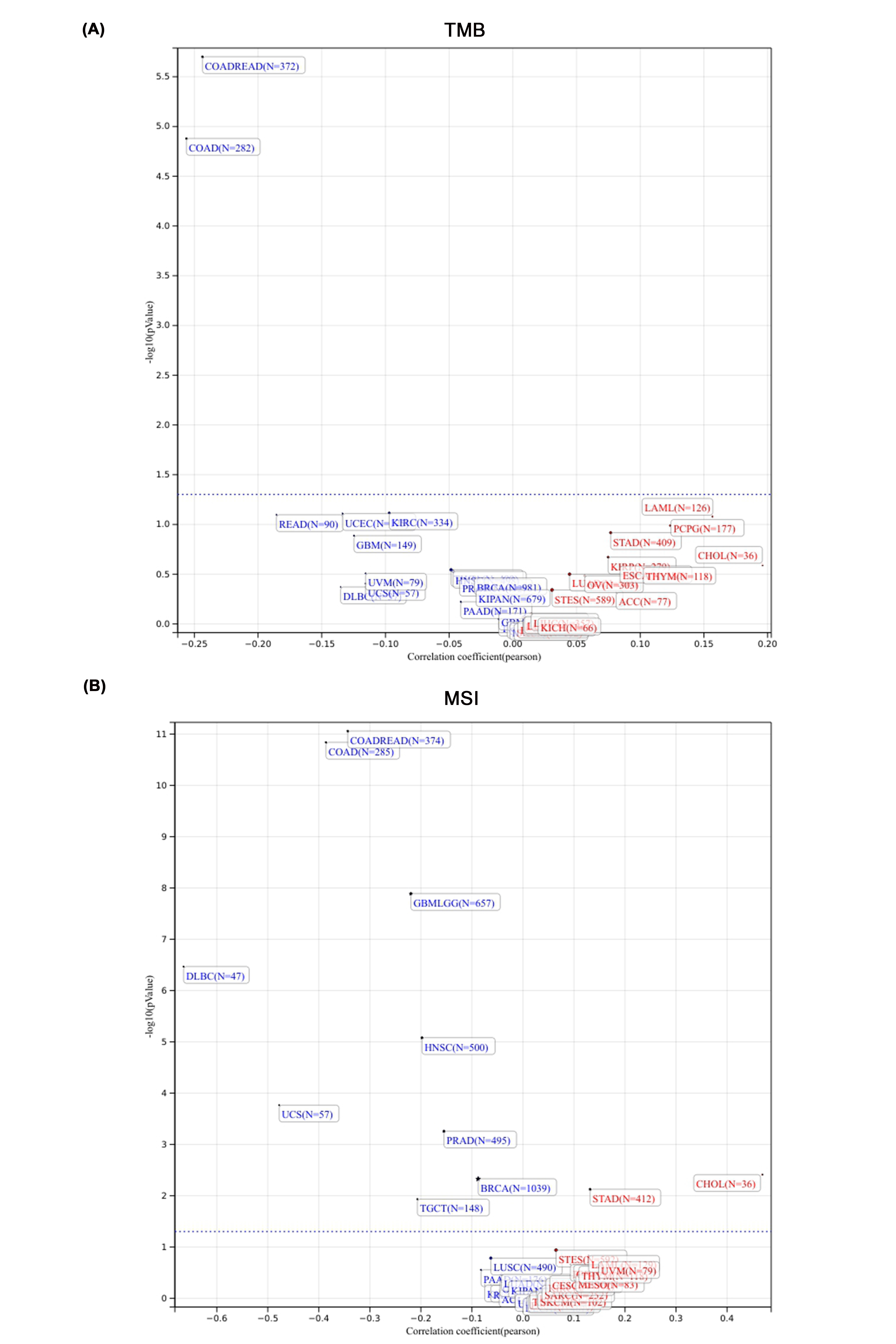


**Supplementary Figure 6 The relationship between the expression level of ZNRF2 and (A)** TMB and **(B)** MSI**.**


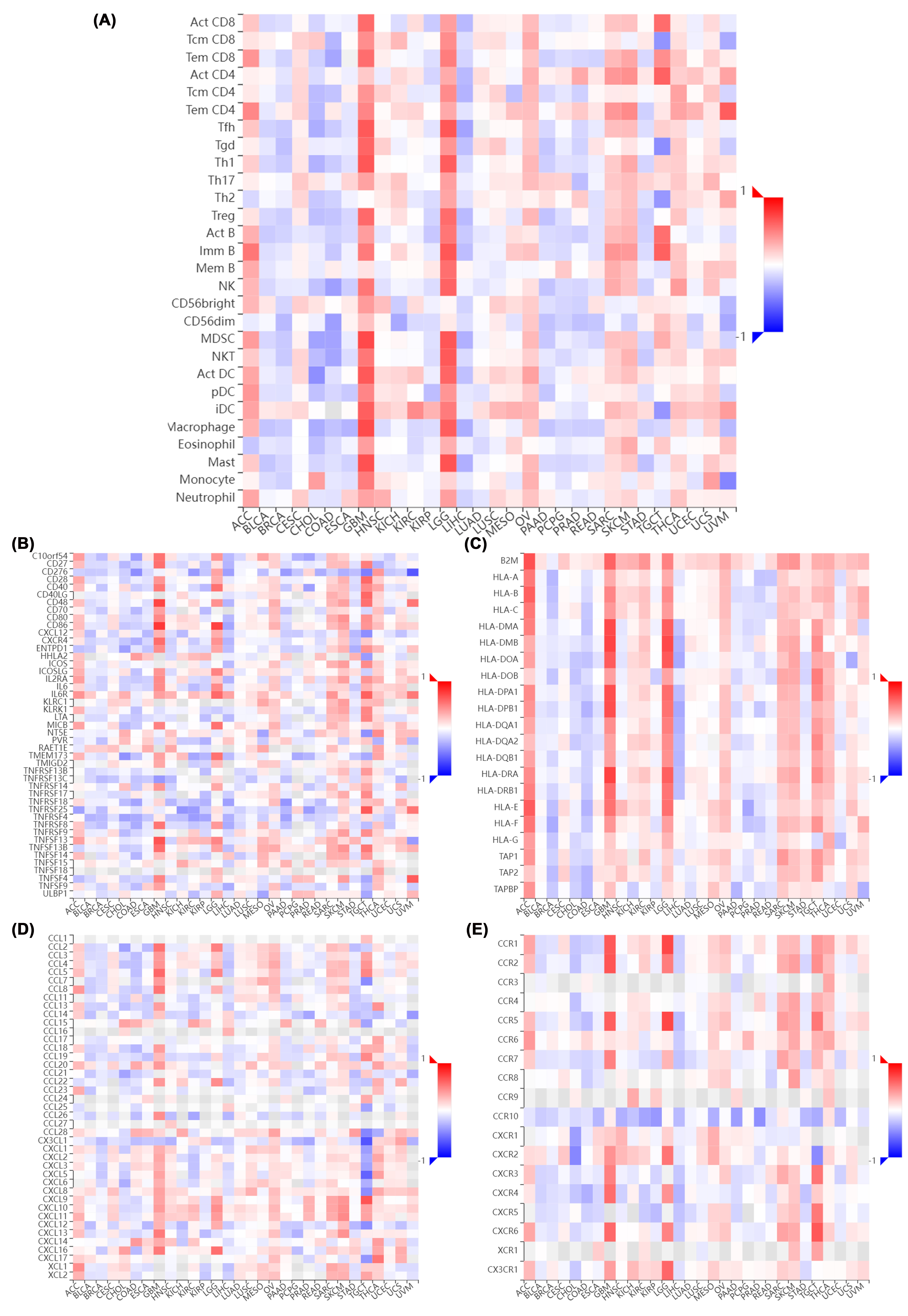


**Supplementary Figure 7. Relationship between ZNRF2 expression levels and tumor-infiltrating lymphocytes, immune-stimulators, MHC molecules, chemokines, and chemokine receptors. (A)** Tumor-infiltrating lymphocytes, **(B)** Immune-stimulators, **(C)** MHC molecules, **(D)** Chemokines, and **(E)** Chemokine receptors.


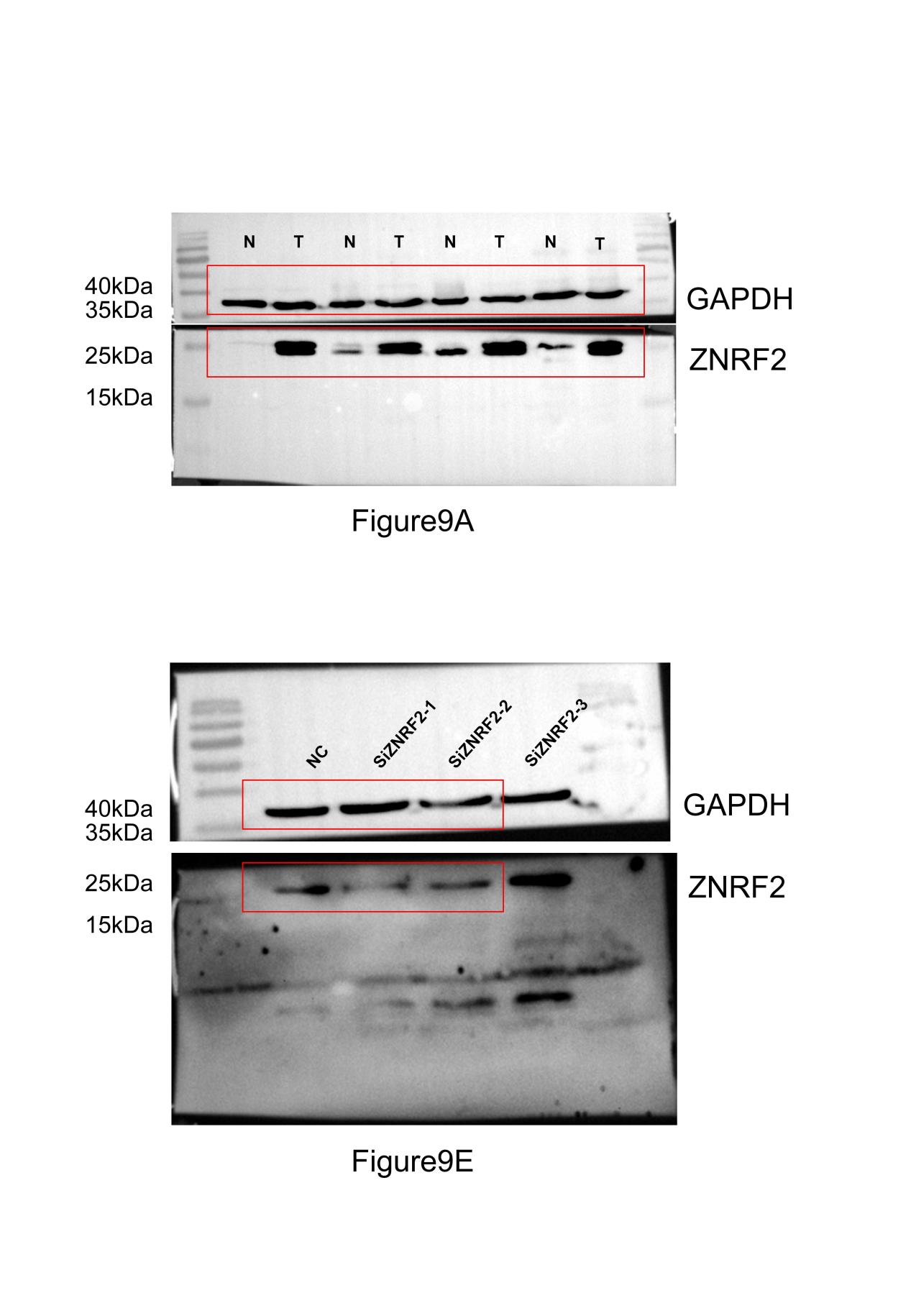


**Supplementary Figure 8.** All the uncropped data of Western Blotting.

**Table S1. Analysis of CPTAC-identified phosphorylation sites of *ZNRF2* via the PhosphoNET database.**


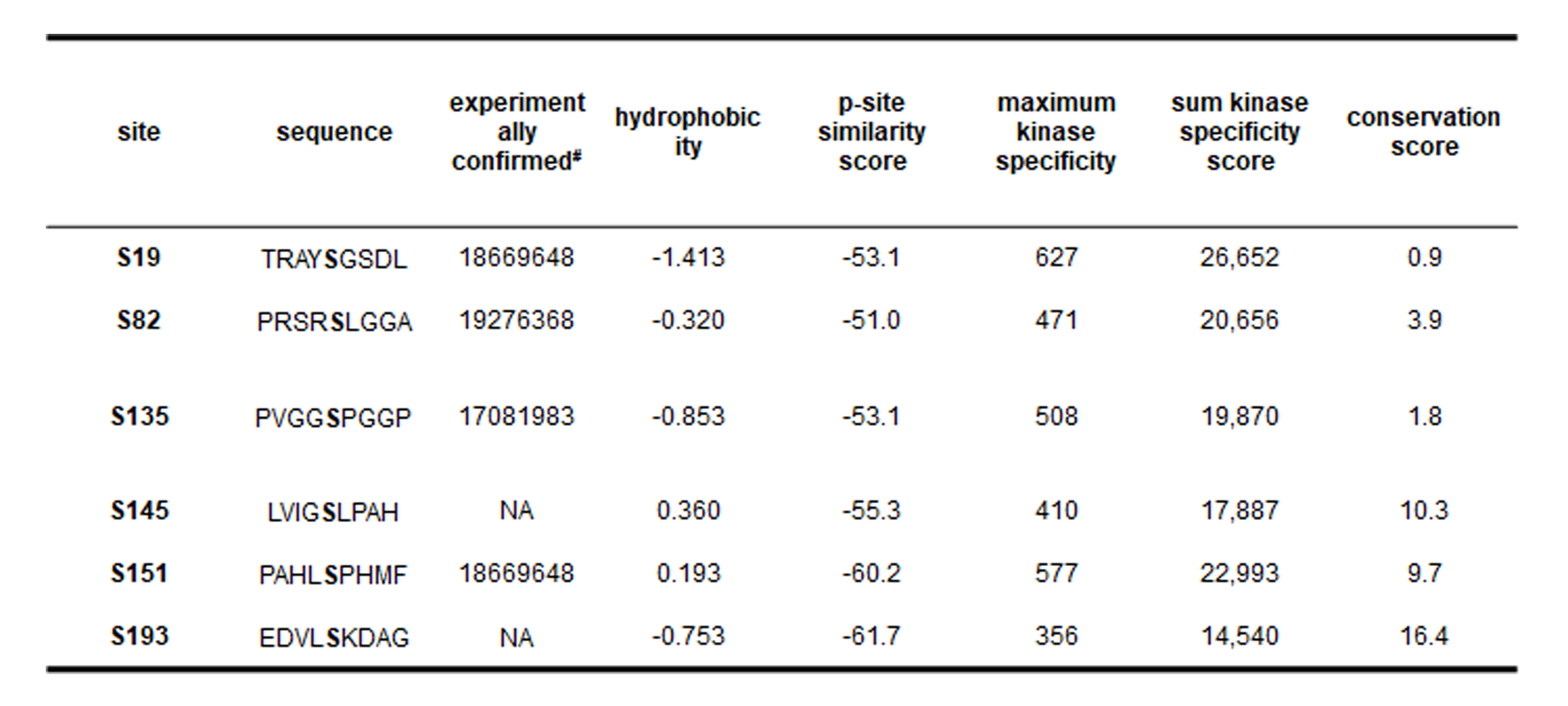


#The PMID (PubMed Unique Identifier) information of the publication was provided; NA, not available.
